# Optical, Dielectric, Magnetic, Photocatalytic, and Antibacterial Properties of Ga-Doped BiGa_*x*_Fe_1–*x*_O_3_ Synthesized by the Microemulsion Approach

**DOI:** 10.1021/acsomega.3c06132

**Published:** 2023-12-16

**Authors:** Zarish Nazeer, Ismat Bibi, Farzana Majid, Shagufta Kamal, Norah Alwadai, Muhammad I. Arshad, Adnan Ali, Shazia Nouren, Maryam Al Huwayz, Munawar Iqbal

**Affiliations:** † Institute of Chemistry, The Islamia University of Bahawalpur, Bahawalpur 63100, Pakistan; ‡ Department of Physics, University of the Punjab, Lahore 54590, Pakistan; § Department of Biochemistry, Government College University, Faisalabad 38040, Pakistan; ∥ Department of Physics, College of Sciences, Princess Nourah bint Abdulrahman University, P.O. Box 84428, Riyadh 11671, Saudi Arabia; ⊥ Department of Physics, Government College University Faisalabad, Faisalabad 38040, Pakistan; # Department of Chemistry, Government College Women University, Sialkot 51300, Pakistan; ¶ Department of Chemistry, Division of Science and Technology, University of Education, Lahore 54770, Pakistan

## Abstract

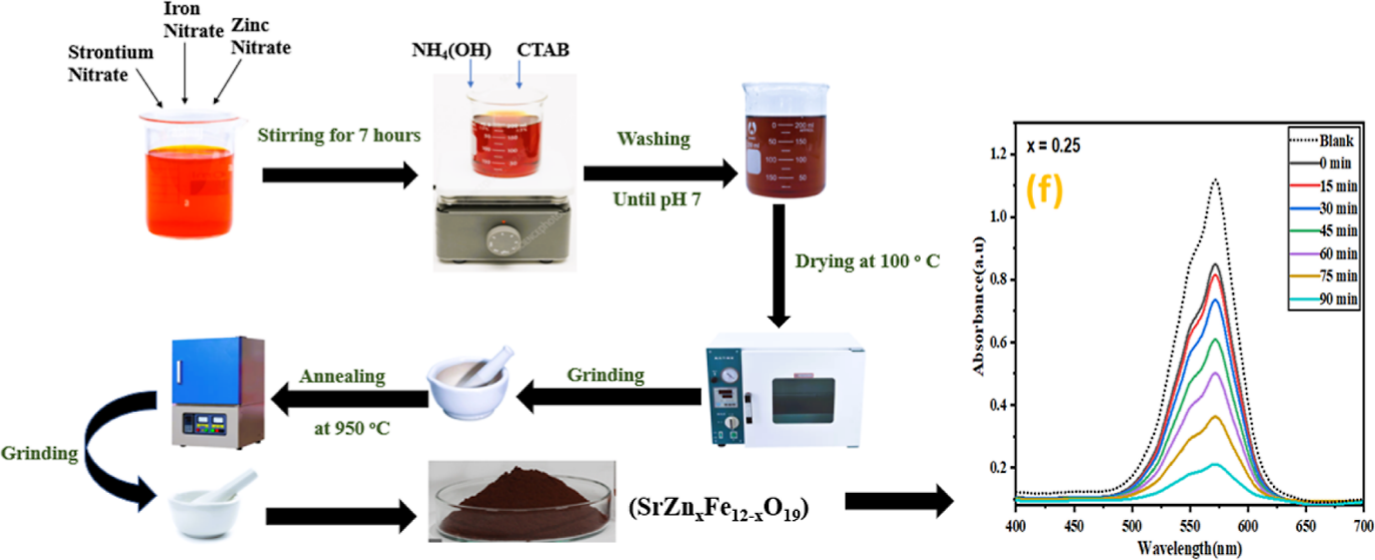

The effect of Ga-substitution on bismuth ferrite BiGa_*x*_Fe_1–*x*_O_3_ (*x* = 0, 0.05, 0.10, 0.15, 0.20, and 0.25) properties was investigated, which was fabricated using a microemulsion route. X-ray diffraction analysis confirmed that specimens had a single-phase rhombohedral structure with space group *R*3̅*c*. The concentration of Ga had an impact on various properties such as structural parameters, crystalline size, porosity, and unit cell volume. The samples exhibited notable values for the dielectric constant, tangent loss, and dielectric loss in the low-frequency range, which declined as the frequency increased due to different polarizations. The increment in the AC conductivity was associated with rise in frequency. The *P*–*E* loops demonstrated that the samples became more resistive as the Ga concentration increased. The retentivity (*M*_r_) and saturation magnetization (*M*_s_) values reduced as the Ga content increased, although all samples had *H*_c_ values within the range for electromagnetic materials. The Ga-substitution had a synergistic effect on the electrochemical characteristics of BiGa_*x*_Fe_1–*x*_O_3_, resulting in greater conductivity than that of undoped BiFeO_3_. These enhanced properties contributed to their higher photocatalytic activity in the degradation of crystal violet under visible light irradiation. The doped BiGa_*x*_Fe_1–*x*_O_3_ exhibited 79% dye degradation after 90 min of illumination compared to 54% for pure BiFeO_3_. Recycling experiments confirmed the stability and reusability of the synthesized nanoparticles. The antibacterial activity of the samples was certified against various microbes, and the doped BiGa_*x*_Fe_1–*x*_O_3_ showed promising activity. Thus, doped materials are good candidates for memories, dielectric resonators, and photovoltaics because of their high dielectric constant and AC conductivity, while their higher photocatalytic activity under visible light makes them promising photocatalysts for removing noxious and harmful effluents from wastewaters.

## 1 Introduction

The modern technology development relies on multifunctional materials, posing new and exciting challenges. Complex oxide materials stand out among the many options available due to their remarkable properties, including ferroelectricity, multiferroicity, high-temperature superconductivity, and magnetoresistance. These unique characteristics make them extremely valuable for diverse applications.^1–3^ The discovery of multifunctional materials has expanded our understanding of the magnetoelectric effect. In oxide materials, a close relationship exists between their properties and their structure, making them particularly sensitive to crystal morphology and various structural conditions. Multifunctional oxides have attained substantial devotion among researchers in modern years owing to their unique properties, containing photovoltaic and photocatalytic capabilities. As a result, they hold great promise for use in photoactive devices.^4,5^ Developing new semiconducting materials with photocatalytic properties has attracted the attention of the researchers. Multiferroic materials attracted considerable devotion due to their unique characteristics, including ferroelectric ordering, electrical tunability, and spontaneous electric polarization. However, identifying perovskites with purely magnetic ferroelectric behavior has proven challenging, as the d orbitals of transition metals are only partially filled.^6–10^

Bismuth possesses a unique characteristic due to the presence of its 6S2 orbitals, which allows it to avoid a common issue in materials with a perovskite-like structure.^11^ Such materials exhibit various properties including antiferromagnetism, superconductivity, ferroelectricity, ferromagnetism, and high dielectric constant.^12,13^ In bismuth ferrite nanoparticles (NPs), multiferroicity arises from the alignment of two perpendicular magnetic and electric components in a cycloidal spinel order, with a periodicity of approximately 62 nm. This arrangement leads to G-type antiferromagnetism, with the magnetic moment of Fe^3+^ being ferromagnetically connected to the (001) planes and antiferromagnetically linked to the neighboring planes.^14^

Studies conducted on bismuth ferrite NPs have indicated that their magnetic properties disappear when the crystallite size exceeds 65 nm, but their multiferroic characteristics dominate at smaller crystallite sizes. Furthermore, higher magnetic moments are perceived in smaller crystalline sizes because of the suppression of the cycloid structure. The existence of oxygen defects has also been detected to enhance Fe^2+^ ions, which increases the magnetic impurity Fe_2_O_3_, thus serving as the source of magnetism in BFO NPs.^15^

Various methods have been employed to synthesize BFO nanomaterials, including the coprecipitation technique, hydrothermal method, autocombustion synthesis technique, microemulsion approach, and sol–gel method.^16–22^ Among these methods, the sol gel technology stands out due to its capability to control chemical compositions and particle sizes. The sol–gel method offers advantage of homogeneous mixing of metals with precursors at molecular and atomic levels, without the formation of any secondary phases.^16^

BiFeO_3_ possesses a deformed rhombohedral perovskite-type structure and belongs to the *R*3*c* space group. However, the literature indicates that intrinsic limitations, including structural instability and significant current leakage, are associated with BFO NPs. Researchers have been actively addressing these limitations by exploring various fabrication techniques and enhancing the multiferroic properties through the substitution of metal ions.^15,23^ In a recent study, Kumar and Singh^24^ investigated Bi_1–*x*_Ca_*x*_Fe_1–*x*_Ti_*x*_O_3_ NPs and discovered that the latent magnetism trapped within the toroidal spin of BFO structure was released, resulting in increased magnetization.

BiFeO_3_ has an *R*3*c* space group and a deformed rhombohedral perovskite-type structure. According to the BFO literature, structural instability and significant current leakage are also listed as intrinsic limitations in BFO NPs.^16–20^ Numerous researchers have worked hard to find solutions to these issues by enhancing the multiferroic properties, for instance, the substitution of rare-earth and metal ions, utilizing various fabrication techniques. In their study of Bi_1–*x*_Ca_*x*_Fe_1–*x*_Ti_*x*_O_3_ NPs, Kumar and Singh^24^ found that the latent magnetism that had been trapped within the toroidal spin of BFO structure had been released, leading to enhanced magnetization. In a study showed by Sun et al.,^25^ it was found that dielectric constant and ferroelectric polarization were improved through substitution of rare-earth oxides, specifically with Tb and Sm rare-earth oxides. Furthermore, multiferroic properties of BiFeO_3_ NPs can also be enhanced by replacing Fe key sites with transition metal ions, i.e., Cr, Ca, and Mn, and eliminating the spin cycloid. Chang et al.^26^ explored the ferroelectric, structural, and electrical properties of Cr-doped BiFeO_3_ NPs, which showed promise in improving MAM characteristics for absorber applications. Therefore, doping is a viable option for improving the properties of the BFO NPs. In extreme conditions, the values of t for the Bi- and Fe-site doping become 0.678 and 0.91, respectively, when trivalent Bi^3+^/Fe^3+^ cations are completely substituted by the isovalent Ga^3+^. In contrast to the structural transition that occurs when Bi is replaced by Ga, there are three benefits when Ga is substituted for Fe: (i) it preserves the initial structure of the material because of similar ionic radii of Ga (0.62 Å) and Fe (0.64 Å), (ii) it disrupts the G-type AFM network and improves magnetization of the BiFeO_3_ system, and (iii) it preserves the ferroelectricity of the material owing to the unspoiled sublattice of Bi and uninterrupted charge balance. Experiments demonstrated that Fe-site doping is possible in Bi-rich environments. At low doping concentrations, Ga effectively takes Fe sites in the doped BiFeO_3_ systems that crystallize in rhombohedral frameworks with the *R*3*c* space group.^27^ Yan^28^ observed an exceptional ferroelectric polarization 230 μC cm^–2^, but Cao^29^ did observe the same trend. Clearly, the precise point of view has not been determined. Thus, in this work, we have investigated the various properties of Ga-doped BFO synthesized by the microemulsion route.

Semiconductor-based photocatalytic processes, for instance, photocatalytic degradation of environmental toxins, photocatalytic water splitting for hydrogen evolution, and photoreduction of CO_2_ to fuel molecules, have become increasingly important in solar energy conversion and environmental purification. Bismuth ferrite (BFO), a perovskite material with a relatively narrow band gap (2.2 eV), intrinsic electric polarization field, strong chemical strength, and low cost, has been proposed as a potential visible light-focused photocatalyst for removing contaminants.^16,18^ However, BFO’s limited carrier mobility and quick recombination of photogenerated e^–^–h^+^ pairs have hindered its photocatalytic efficiency. To enhance BFO’s photocatalytic activity, various methods have been developed, including cocatalyst loading, structural control, heterostructure assembly, and alien metal ion doping.^17,18,30,31^ Among these methods, doping BFO with foreign metal ions at the A or B site of the ABO_3_ lattice is considered one of the most efficient approaches to increase photocatalytic efficiency. Doping creates electron or hole trapping sites within the photocatalyst lattice, which enrich the formation of excited e^–^–h^+^ pairs, resulting in enhanced photocatalytic capability of BFO.^16,32,33^

In this study, the main objective was to investigate the potential of a novel composition, BiGa_*x*_Fe_1–*x*_O_3_, for use in photocatalytic applications. The composition included varying concentrations of gallium (Ga) dopant, with *x* ranging from 0 to 0.25. First, crystal arrangement of the fabricated nanomaterial was examined using the X-ray diffraction (XRD) technique. The study aimed to determine whether the doping of gallium had any impact on the crystal structure of the BFO material. The magnetic parameters of the material were also studied to examine the impact of substitution on magnetic properties of the material. The study also analyzed the optical band gap of the material and how this could impact its photocatalytic activity. The catalytic potential of the material was assessed by measuring its capability to destroy noxious waste under visible irradiation. Overall, this study aimed to investigate the potential of BiGa_*x*_Fe_1–*x*_O_3_ as a photocatalyst for environmental remediation applications. By investigating structural, optical, and magnetic properties of the material, researchers hoped to gain a better understanding of how the material could be optimized for photocatalytic applications.

## 2 Materials and Methods

### 2.1 Chemicals and Reagents

The metal salts, i.e., Bi(NO_3_)_2_·6H_2_O, Ga(NO_3_)_3_, and Fe(NO_3_)_3_·6H_2_O, were precured from Sigma-Aldrich, while NH_4_OH, CTAB, and crystal violet (CV) dye were acquired from Merck. Stock solutions and dilutions were prepared with distilled water throughout the whole investigation.

### 2.2 Synthesis of BFO and BiGa_*x*_Fe_1–*x*_O_3_ NPs

**Figure 1:**
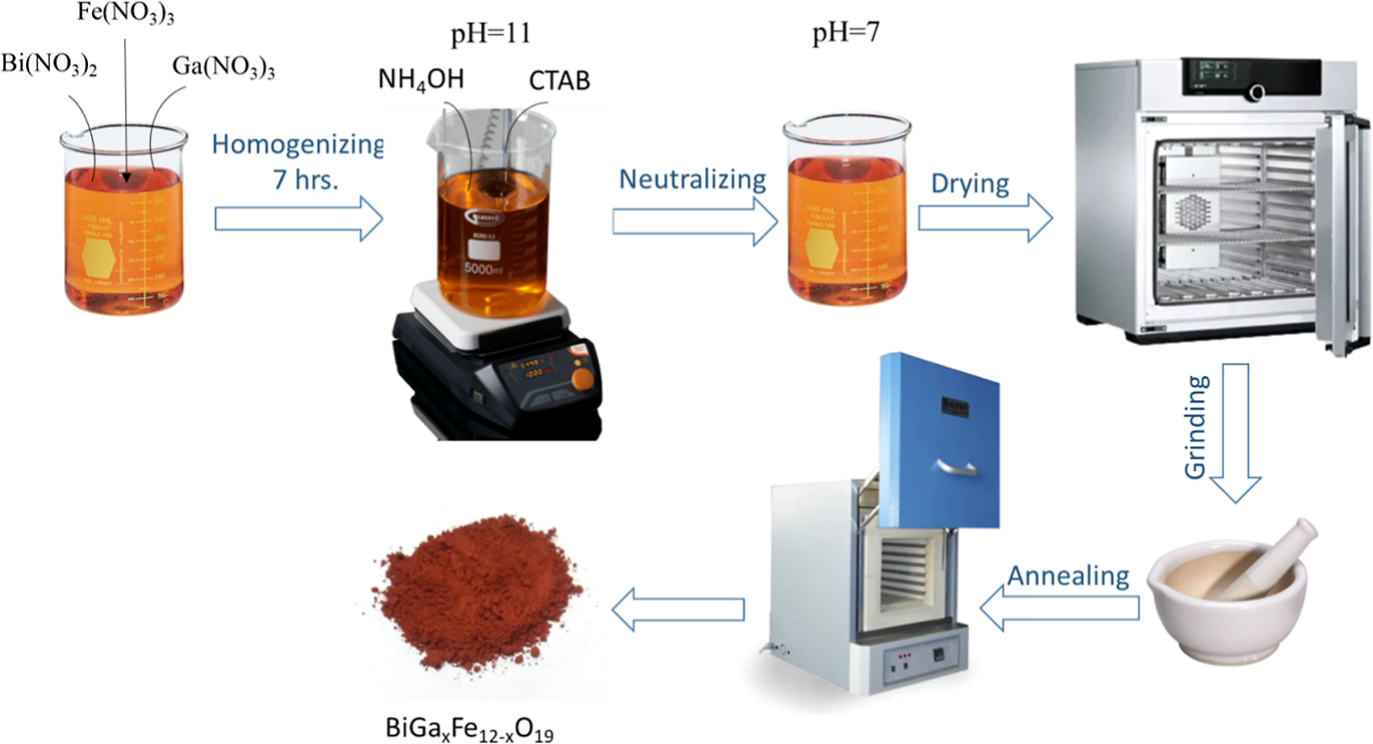
Synthesis scheme of BiGa_*x*_Fe_12-x_O_19_ via a microemulsion route (photograph courtesy of “Zarish Nazeer”. Copyright 2023).

To prepare pure BiFeO_3_ and BiGa_*x*_Fe_1–*x*_O_3_ NPs (where *x* = 0.00, 0.05, 0.10, 0.15, 0.20, and 0.25), a protocol was followed. The metal nitrates (stoichiometric ratio) were dissolved in water to form the required solutions. A 500 mL beaker was taken, and solutions were mixed and then heated at 60 °C temperature for about 30 min. The heat was turned off after the desired temperature was reached but stirring was continued. The pH was adjusted to 11 by using an ammonium hydroxide solution. The solution was then stirred on a magnetic hot plate stirrer for 7 h. The solution was washed repeatedly until the pH was neutral. The resulting precipitates were then washed multiple times with deionized water and then dried for 12 h at 150 °C. The oven-dried samples were ground to fine powder and calcined for 6 h at 950 °C (Figure 1). Equations 1 and 2 describe the proposed reactions for the synthesis of BFO formation.
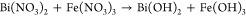
1
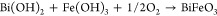
2

### 2.3 Characterization

A range of systematic techniques were employed to examine the nanomaterials produced using the microemulsion technique. Crystallinity analysis was accomplished by means of X-ray diffraction (Philips X-ray Pert PRO 3040/60), while the M-S Radiant instrument (USA) was utilized to evaluate ferroelectric properties via *P*–*E* loop tracing. The chemical bonding between the elements was verified by utilizing FTIR (Nexus-470-spectrophotometer) and Raman spectroscopy. The dielectric properties were measured using a Waynker WK6500B LCR meter for the frequency range of 20 Hz to 0.2 GHz, and an *I*–*V* (current–voltage) measurement was recorded using a Keithley 2400 m. Finally, UV–vis experiments were carried out using a Carry-60 spectrophotometer.

### 2.4 Photocatalytic Activity

Both BiFeO_3_ and BiGa_*x*_Fe_1–*x*_O_3_ photocatalytic potentials were appraised for CV dye under visible light. Throughout the photocatalytic experiment, 15 mg of the photocatalyst was mixed to a solution of CV dye (150 mL, 10 mg/L). The mixture was positioned in the dark under stirring for 20 min and then subjected to a 160 W Xe lamp. Required number of samples were collected from the dye solution at predetermined time intervals and at 588 nm absorbance was recorded using a Carry-60 spectrophotometer. Before each absorbance measurement, the solution was centrifuged to remove the catalyst powder. The CV concentration was determined from the absorbance values, and the percentage degradation was estimated using eq 3, where A_o_ and A_t_ represent the absorbance values at time “0” and time “*t*”, respectively.
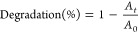
3

## 3 Results and Discussion

### 3.1 XRD Analysis

**Figure 2:**
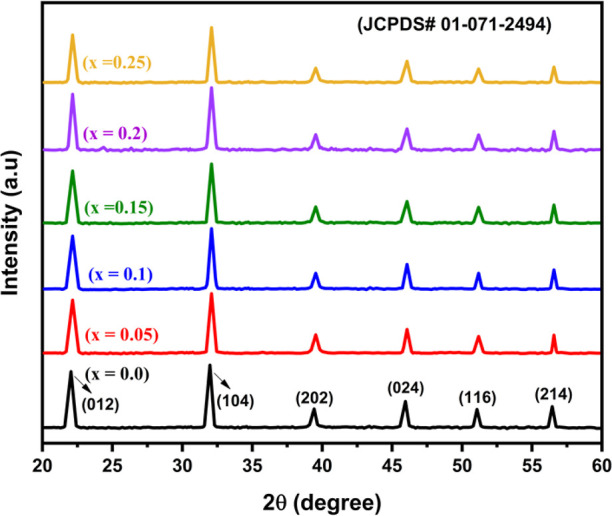
XRD patterns of BFO and BiGa_*x*_Fe_1–*x*_O_3_ NPs.

Figure 2 exhibits the XRD patterns of BiGa_*x*_Fe_1–*x*_O_3_ (BFOG) NPs with various Ga doping levels (*x* = 0.0, 0.05, 0.10, 0.15, 0.20, and 0.25) at room temperature (298 K). The lattice parameters, average grain sizes, and crystal structure of the nanocrystalline BFOG are summarized in Table 1. All the XRD peaks match suitably with the standard (JCPDS file no. 71-2494), indicating the perovskite structure formation. The specific peaks at 2θ = 22.3, 31.7, 39.4, 45.6, 51.2, 56.3, and 56.8° correspond to the (012), (104), (202), (024), (116), (018), and (214) planes, respectively. The XRD patterns approve nearly single-phase rhombohedral structure of the synthesized samples, which can be described using the *R*3*c* space group in a hexagonal reference frame. No secondary impurity peaks, such as Bi_2_Fe_4_O_9_, Fe_2_O_3_, Bi_24_Fe_2_O_39_, and Bi_2_O_3_, were observed in the pristine or doped NPs with *x* ≤ 0.25.

**Table 1:** Ga Doping Influence on Different Structural Parameters of BiGa_*x*_Fe_1–*x*_O_3_ NPs

doping content *x*, *y*	0.0	0.05	0.1	0.15	0.2	0.25
cell volume (A)^3^	382.8	380.7	380.2	378.9	378.8	377.1
lattice constant *a* (Å)	5.70	5.68	5.65	5.63	5.62	5.61
lattice constant *c* (Å)	13.92	13.89	13.88	13.86	13.85	13.82
*c*/*a* ratio	2.44	2.44	2.45	2.46	2.46	2.46
crystallite size (nm)	34.5	32.6	31.04	30.3	29.7	29.2
X-ray density (g cm^–3^)	2.71	2.72	2.73	2.75	2.76	2.78
bulk density (g cm^–3^)	1.40	1.38	1.35	1.32	1.26	1.14
porosity	48	49	51	52	54	59
dislocation density	1.18	1.13	1.06	1.02	0.99	0.91
strain	0.496	0.488	0.487	0.476	0.474	0.471

Table 1 presents the lattice parameters, c/a ratios, and average grain sizes of BFOG NPs. The doping concentration of Ga^3+^ ions decreases lattice parameters *a* and *c*, which are consistent with reports (*a* = 5.587 Å and *c* = 13.860 Å). However, the *c*/*a* ratios of the different doping levels are all 2.44. As the Ga content increases, the lattice parameters gradually compress, causing the ferrite structure to shrink. This reduction in structural size may be accredited to the replacement of Fe^3+^ (ionic radius of 0.64 Å) with Ga^3+^ ions (ionic radius of 0.62 Å). The Ga–O bond in the BiFeO_3_ host is stronger than the Fe–O bond due to the lower electronegativity of Ga than Fe (1.81 vs 1.83 on the Pauling electronegativity scale), which may account for the observed trend. The cell volume of BiFeO_3_ decreases upon Ga^3+^ ion doping. These findings suggest that the doping causes changes in the rhombohedral BiFeO_3_ structure, such as the replacement of Fe with Ga in the parent structure. The Fe–O–Fe bond angle and the Fe–O bond length may also shift with Ga substitutions as the ionic radius of Ga^3+^ at octahedral sites is smaller than that of Fe^3+^. Moreover, as Ga concentration increases to *x* = 0.15, the XRD peaks shift slightly to higher 2θ values, accompanied by a decrease in peak intensities. This observation indicates that Ga-substitution distorts the rhombohedral structure, which is also reported for other rare-earth-substituted BFO ceramics.^34,35^ The average particle size of pristine and all Ga^3+^-doped BiFeO_3_ NPs was found to be between 29 and 34 nm by using the Debye–Scherer relation (eq 4).
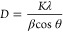
4Here, β represents the angular line width at maximum intensity and θ denotes Bragg’s angle of peak intensity.

The lattice unit cell volume (*V*_cell_) and lattice constants (*a*, *c*) were obtained using eqs 5–8, where “*M*” denotes the molecular mass, “*Z*” denotes the formula unit number of the hexagonal crystal system, NA is Avogadro’s number, ρ_m_ is the bulk density, ρ_x_ is the X-ray density, and P is the porosity (%). Table 1 shows an increase in both bulk density and X-ray density with increasing the doping concentration of Ga. The increase in bulk density can be attributed to a higher concentration of Ga in comparison to Fe.
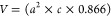
5
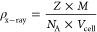
6
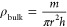
7
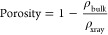
8

### 3.2 FTIR Spectroscopic Analysis

**Figure 3:**
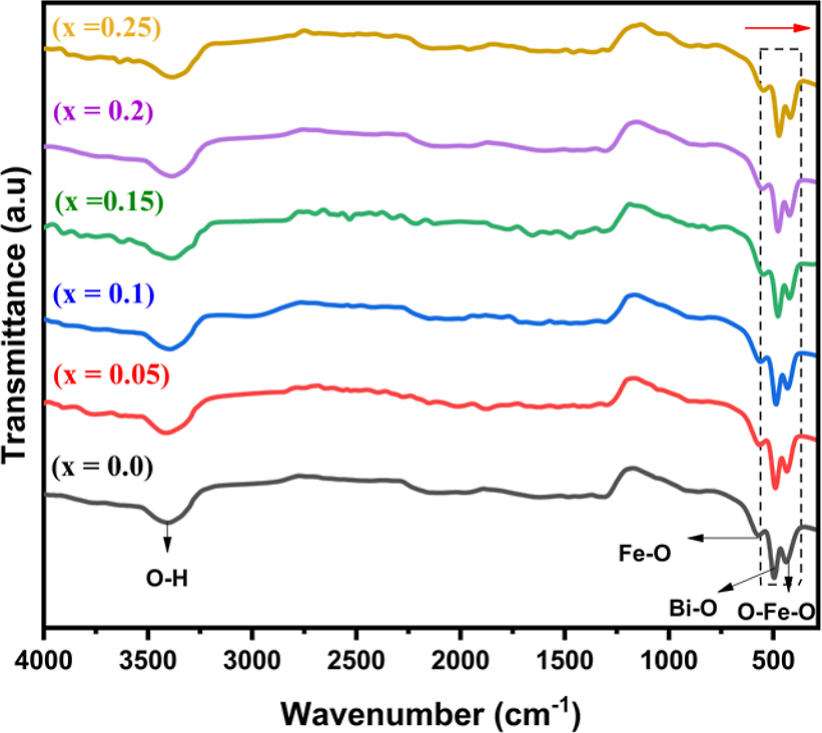
FTIR spectra of different compositions of BiGa_*x*_Fe_1–*x*_O_3_ NPs.

The FTIR spectra between 400 and 4000 cm^–1^ have been used to characterize pure and Ga-substituted BiFeO_3_ NPs and are shown in Figure 3. The bending/curved vibrations of the O–Fe–O bond in the octahedral form of the FeO_6_ unit is indicated by an absorption edge at 440–450 cm^–1^, which is designated as E(T0_8_). E(T0_9_) and A_1_(TO_4_) modes were separated into two groups for the region between 480 and 680 cm^–1^. The elongated vibrations of the Fe–O bond in FeO_6_ is what causes the absorption band mode to be around 575 cm^–1^, and the vibrational mode of the Bi/Ga–O bond is near 550 cm^–1^. The shifting in the active IR modes of various samples can also be seen in the FTIR spectra (marked by the red arrow). From Figure 3, which presents a set of FT-IR spectra spanning the wavenumber range of 300–4000 cm^–1^, one can clearly notice a shift in the location of the spectra. The absorption peak shifts slightly toward a lower wavenumber by increasing the Ga concentration; this could be due to Ga’s smaller ionic radius when compared to Fe. Furthermore, this shift indicates bending of Bi–O and Fe–O bonds caused by octahedron deformation with the Ga-substitution.^36^ Owing to antisymmetric and antisymmetric stretching of H_2_O and hydroxyl ions, respectively, each sample displays a broad absorption band in the range of 3300–3600 cm^–1^.

#### 3.2.1 Raman Spectroscopic Analysis

**Figure 4:**
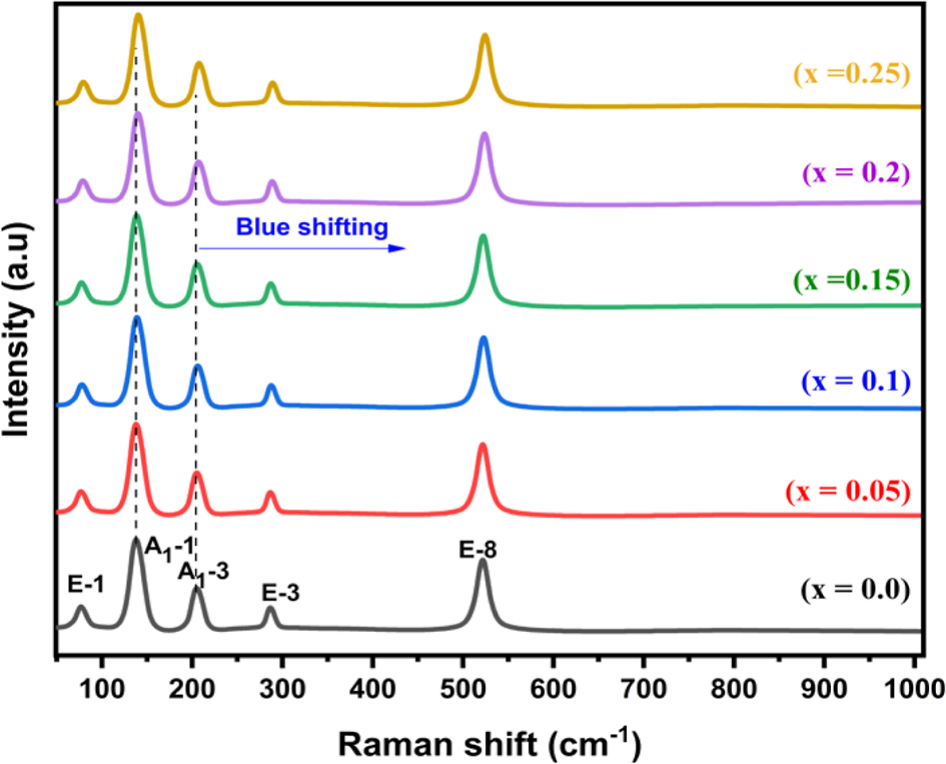
Raman spectra of BFO and BiGa_*x*_Fe_1–*x*_O_3_ NPs.

Raman studies on doped BiGa_*x*_Fe_1–*x*_O_3_ NPs have also been conducted in order to gain more understanding of spin phonon interaction and lattice properties.^37^Figure 4 compares the Raman spectra of undoped and doped BFO NPs at RT by the parallel polarization with laser having an exciton wavelength (λ) of 488 cm. The changes in bond length among atoms in the BFO lattice brought on by doping is what causes the Raman peaks in doped NPs to shift in relation to pristine BFO NPs.^37^ Due to the identical crystal structure, the same Raman phonon modes are displayed by both the pure and doped NPs in this case. Based on a group theoretical study, it is predicted that pristine BFO materials will have 13 (4 A_1_ + 9 E phonon modes) Raman modes.^38^ Experimentally, all 13 Raman active modes for the bulk BFO materials have been identified, with only minor variations in peak positions resulting from changes in the disorder and oxygen bonding.^37^ Each peak position’s reported natural frequency (cm^–1^) can be ascribed to the separate Raman-active mode here. Table 2 compares the positions of Raman modes of pristine BFO NPs to previously reported data on BFO NPs.

**Table 2:** Location of BFO NP Raman Modes (cm^–1^) in our Research Compared to Previously Reported Values

Raman modes	present study	Fukumura et al.^39^	Jaiswal et al.^40^
A_1_-1	137	147	139
A_1_-2	158	176	169
A_1_-3	205	227	216
A_1_-4		490	425
E		265	260
E	286	279	276
E		375	348
E		437	467
E	522	473	529
E	76	77	72

In our study, for BiGa_*x*_Fe_1–*x*_O_3_ NPs (Figure 4), the two peaks at 137 and 205 cm^–1^ can be classified as longitudinal optical (A_1_) phonon modes A_1_-1 and A_1_-3, accordingly. The following three peaks, located at 76, 286, and 522 cm^–1^, are connected with transverse optical (E) phonon modes.^37^ Some peaks (absence) could be attributed to increased local stress in NPs and intrinsic defects in the doped NPs. Since the material is polycrystalline, the spectra described here are for parallel polarization.^37^ The magneto–electric coupling in samples is caused by the Bi–O_1_ vibrational peak at 137 cm^–1^. As the dopant concentration increased, it is observed that the Bi–O_1_ peak (A_1_-1 mode) shifts toward a higher frequency, which may be the result of a change in the force constant in doped NPs brought on by the microstructural modifications associated with doping. The ongoing decline in peak intensity with increasing dopant content suggests the inhibition of the contributions of Bi–O_1_ vibrational mode, which is likely instigated by enhanced coupling between magnetic order and ferroelectric parameters as a consequence of quantum confinement.^37^

### 3.3 Current Voltage (*I*–*V*) Analysis

In order to determine the consequences of Ga doping on the electrical conductivity of the doped material, two probe *I*–*V* measurements were carried out in the 20 to −20 V range using pristine and doped BiGa_*x*_Fe_1–*x*_O_3_. Pellets with a consistent diameter of 820 mm and width of 2.16–2.31 mm were synthesized, and their surface area was calculated using the formula shown in eq 9.
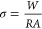
9

The (*I*–*V*) of the doped sample shows a linear response, while the (*I*–*V*) of pure BiFeO_3_ has an inverse S shape, indicating a semiconductive behavior (Figure S1a–c). The current–voltage relationship of the doped sample remained nearly linear as the concentration of the dopant was increased. Ga doping was shown to be responsible for this behavior, which is indicative of high EC in comparison to BiFeO_3_. Specifically, Ga^3+^ ion takes the place of Fe^3+^ ion in the perovskite materials octahedral positions, respectively. When compared with the Fe ions in the BiFeO_3_ sample, the EC (*I*–*V*) behavior of Ga was encouraging.

### 3.4 Electrical Polarization

Ferroelectric study of BiGa_*x*_Fe_1–*x*_O_3_ was carried out using pallets with a diameter of 7 mm that were covered in silver paste serving as the electrode. A PUND (positive up and negative down) mode was used to detect the induced polarity in order to make it easier to subtract nonferroelectric components such as leakage current and space charge. For the perovskite ferroelectrics functioning at high temperatures, current leakage is a significant issue that makes measuring the hysteresis loop at high temperatures challenging. The room-temperature measurements of the pristine BFO and BiGa_*x*_Fe_1–*x*_O_3_ polarization field hysteresis loops are shown in Figure S2. All of the polarization loops are concave-shaped and exhibit intrinsic ferroelectric behavior. The *P*–*E* loops in each of the compositions were altered by doping of substituents into the perovskite structure. The curves progressively begin to round up at both negative and positive field regions, increase in polarization in the positive region of the electric field, and decline in polarization in the negative region. This phenomenon may be the result of an escape or destabilization at the negative pole region^41^ altering their characteristic to lossy trends. The pristine sample exhibits a lossy characteristic, and it has been noticed in the doped samples that this response decreases as the Ga^3+^ concentration rise, indicating an increase in the resistive nature of the material owing to accumulation of additional charge carriers at the interface.^42^ The influence of replacement of Fe with Ga on polarization is comparatively weak as the stereochemically active Bi-6*s* lone pair is primarily accountable for ferroelectricity of the bulk material; therefore, polarization is reduced slightly on doping. Table 3 displays the computed values of remanence polarization (*P*_r_), saturation polarization (*P*_s_), and *P*_s_/*P*_r_ for every concentration. For pure BFO, a broader hysteresis loop with a *P*_max_ value of 3.13 × 10^4^ C/cm^2^ was observed. This loop eventually transformed to a narrow shape loop with a *P*_max_ value of 14.7 × 10^4^ C/cm^2^. It has been demonstrated that the value of *P*_r_ for all samples is less than *P*_s_, which is the recognized explanation for the lossy characteristic of the *P*–*E* loops. The substituted samples exhibited lossy characteristics because of hopping electrons among Fe^2+^ and Fe^3+^ ions. Moreover, the replacement of Ga^3+^ for Fe^3+^ at the Fe–O octahedral site resulted in a decrease in *P*_s_ and *P*_r_ values because of off center movement of the Fe^3+^ ions.^41^ The stereochemically active Bi6s lone pair is mainly accountable for ferroelectricity of the bulk material; hence, the effect of replacing Fe with Ga on polarization is quite minor. Ga can inhibit the conversion of Fe^3+^ to Fe^2+^, minimizing conductive losses sufficiently.^43^ Although doping reduces polarization marginally, the projected values are still greater than those of renowned ferroelectrics, i.e., PbTiO_3_ (75 mC/cm^2^) and PbZr_0.52_Ti_0.48_O_3_ (54 mC/cm^2^)^44^.^45^ Based on these results, BiGa_*x*_Fe_1–*x*_O_3_ appears to be the potential material for usage in ferroelectric devices.^46^

**Table 3:** Variation in Ferroelectric Parameters of BiGa_*x*_Fe_1–*x*_O_3_ (*x*, *y* = 0.0–0.25) NPs with an Increase in Dopant’s Content

doping content	composition	*P*_r_ (μC·cm^2^ × 10^–4^)	*E*_c_ (kV/cm)	*P*_s_ (μC·cm^2^ × 10^–4^)
0.0	BFO	6.58	2.45	5.40
0.05	BFOG1	4.98	2.37	4.25
0.05	BFOG1	4.98	2.37	4.25
0.15	BFOG3	3.48	1.95	2.90
0.20	BFOG4	3.01	1.70	2.36
0.25	BFOG5	2.51	1.51	1.86

### 3.5 Dielectric Properties

The dielectric characteristics versus frequency for BFO and BiGa_*x*_Fe_1–*x*_O_3_ were investigated at room temperature utilizing PPC in line with precision using a LCR meter. The silver-coated ends of the fabricated sample served as electrodes, resulting in PPC arrangement. The material to be studied was placed within the sample container before scanning in the range of 100 Hz–10 GHz. The signal presented capacitance (*C*_p_), energy dissipation factor, and impedance, which were used to measure all dielectric parameters.

#### 3.5.1 Dielectric Constant and Dielectric Loss

Equation 10 was used to compute dielectric constant (ε′) values.
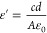
10where *d* and *C* indicate the thickness of the pallet and capacitance, respectively, and *A* and ε_0_ depict the permittivity of the area and free space of pellets. Tangent loss (tan δ) was estimated by eq 11.
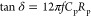
11where parallel equivalent resistance is shown by *R*_p_ and parallel equivalent capacitance and applied field are shown by *C*_p_ and *f*, respectively. Dielectric loss (ε″) was calculated using eq 12.
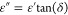
12

The impact of substituents on the dielectric characteristics of BiGa_*x*_Fe_1–*x*_O_3_ is shown in Figure S3. The results showed that values of the dielectric constant were higher in the lower frequency zone; however, the values decreased as the applied field frequency increased, and in the high-frequency range, these values were eventually independent of field. At first, the decline rate was quick, but later, it became slower at higher frequencies. At 100 Hz, the dielectric constant’s maximum value was recorded.

The dielectric behavior observed can be described by Koop’s phenomenological model, which proposes that a dielectric medium comprises conducting grains separated by nonconducting grain boundaries. In this model, high dielectric values detected at low frequencies are attributed to the presence of grain boundaries. The presence of grain boundary defects, interfacial gaps, oxygen vacancies, and dislocation pile-ups in the nanomaterial can lead to space charge polarization, which contributes to a significant increase in the dielectric constant at lower frequencies. It should be noted that the specific mechanisms responsible for the observed dielectric performance may vary depending on the material and experimental conditions. The explanation provided above is based on the general principles of Koop’s phenomenological model and the effects of grain boundaries and associated defects on dielectric properties.^16^

The interfacial polarization mechanism proposed by Maxwell–Wagner can provide a good explanation for the higher value of ε′ at low value of frequency. According to this mechanism, an additional polarization known as interfacial-type polarization is produced at the ferromagnetic and ferroelectric interface when the electrical conductivities of the two materials differ noticeably. This polarization aids in raising the dielectric constant values. At low frequencies, a dielectric material’s dipoles actually do follow the applied field. Since the reaction of interfacial polarization to the applied frequency was somewhat weak, it was further prevalent in regions of low field because charge carrier elements in the dielectric medium require some time to align with the AC field. Ionic and electronic polarizations are the result of structurally deforming a material, whereas relaxing the dielectric dipoles produced orientation and interfacial polarizations. The possibility of electrons reaching grain boundaries is decreased by increasing the frequency of the applied field as a result of a reduction in the electron hopping behavior. Consequently, the dielectric dipoles might not be capable of following the field and may lag behind the electric field, which will lead to a decrease in ε_r_ values. Therefore, it may be assumed that at higher frequency, only electronic polarization arises that causes a reduction in dielectric values.^24^ At that point, all polarizations disappeared, but electronic polarization became more prevalent. Similar declining trends can be seen for tan δ and ε′ in relation to frequency, which could be explained by a drop in the response of electron hopping of dipoles. Multiferroic often exhibit reduction in all of the dielectric properties within the field. This low-leakage current revealed by low dielectric and low tangent loss makes it operative in radio and different higher frequency operated materials.^47^

#### 3.5.2 AC Conductivity

The influence of Ga ion substitution on AC conductivity of doped BiGa_*x*_Fe_1–*x*_O_3_ as a function of frequency is depicted in Figure S4. In the low-frequency range, constant values of AC conductivity were recorded for all samples, which improved with frequency. As a result of grain size boundaries, the electrons may have a slow rate of hopping from one interstitial site to another, using less energy in the process. This could explain the constant value of AC in the lower frequency zone. At grain boundaries, Fe^2+^–Fe^3+^ charge carriers likely exhibit higher electron hopping during the high-frequency range, which may actively contribute to an increase in σ_AC_. The rise in AC conductivity that can be seen in the high-frequency zone may also be the result of reorientation phenomenon, relaxation impact, and dispersion.

#### 3.5.3 AC Resistivity

The relationship between AC resistivity and applied field frequency at room temperature (298 K) for pure and doped BFO compositions is displayed in Figure S5. The AC resistivity (ρ_AC_) for fabricated samples has been significantly decreased up to 10 MHz and then becomes constant. Under applied frequency, the constant amount of resistance observed at ≥10^3^ Hz describes the potential discharge of interfacial-type dispersal or development at the boundary area of unified phases in the sample. The simple principle at work is most likely consistent reactance values over grain boundaries, which facilitated charge carrier hopping at higher frequencies (>10^3^ Hz). As a result, lower reactance values suggested a higher loss of the resistance of doped materials. Such behavior is commonly observed in composites because of the presence of an interfacial dispersion process, which causes the discharge of electrical charges stored up inside piezoelectric granules by the low-resistance path of neighboring BFO particles.^48^

### 3.6 Magnetic Properties

Figure S6 displays M–H loops for both pure and BiGa_*x*_Fe_1–*x*_O_3_ ferrite samples. From the loops, various characteristics counting *M*_r_ (remnant magnetization), *H*_c_ (coercivity), and *M*_s_ (saturation magnetization) were determined. The shape and diameter of the loops are influenced by variables such as chemical composition, distribution of cations, porosity, particle size, etc. The width of loops grows uniformly, indicating an increase in *H*_c_ proportional to the quantity of Ga-substitution (*x*). Coercivity is a process that is influenced by both the material’s internal and external characteristics. Chemical composition and crystal structure determine intrinsic qualities including *M*_s_, anisotropy energy *E*_k_, *T*_c_, and anisotropy field *H*_a_. Extrinsic qualities are those connected with bulk material’s microstructure, including grain shape, grain size, and defects. Magnetic materials that are soft involve a low coercive field. The coercivity value is within a few hundred oersteds, making it appropriate for longitudinally magnetic storage media.^49^ In addition, a low coercivity value is one of the requirements for the EM materials.^50^ Results further indicate that an increase in Ga concentration results in decreasing the exchange coupling, leading to a reduction in both *H*_c_ and *M*_r_ values. The drop in saturation magnetization (*M*_s_) can be attributed to reversible hard displacement or grain boundary motion in the direction of the applied field. This movement can occur only if the domain wall pinning energy is higher than that of the externally applied magnetic field. Therefore, greater displacement results in lower saturation magnetization. Additionally, the compensatory magnetization of two interstitial sites can be expressed as M14 (*M*_b_*M*_a_), where *M*_b_ and *M*_a_ are the magnetizations due to tetrahedral A-sites and octahedral B-sites, respectively. In the result, replacement of Ga^3+^ ions at the B site leads to a decrease in M since the doping Ga^3+^ ions preferentially occupy the B-site.^51^ The magnetic moment of Fe^3+^ ions is greater than that of the nonmagnetic Ga^3+^ ions. As the amount of Fe^3+^ ions at the B-site declined, so did magnetization of the B-substrate, leading to a reduction in saturation magnetization (*M*_s_) of Ga-doped samples. The decline in *M*_s_ and *M*_r_ with increasing substitution could also be attributable to spin canting and colinearity breakage.^19^ The magnetic characteristics of bismuth ferrites were clearly impacted by the introduction of Ga-substitution.

### 3.7 Photoluminescence Property

Third optical properties rely on the transitions between the O 2p and Fe 3d orbitals, while the PL (photoluminescence) is attributed to the e^–^–h^+^ pair recombination. These processes are regulated by the Pauli exclusion principle.^52^ The formation of photogenerated charge carriers is a key factor in the PCA (photocatalytic activity) of semiconductor catalysts, and this rate can be investigated by studying the PL. The PL spectra of undoped and doped BFO are displayed in Figure S7. The doped compositions showed higher PL intensities when compared to pristine BFO. The increase in the PL intensity can be seen to be consistent with increasing dopant concentration. This increase in PL intensity appears to be associated with either enhancement of electron hole recombination or reduction in electrical charge separation caused by the addition of dopants to the perovskite structure. The highly doped sample had the higher intensity PL peak, related with the diminution of optical activity among O 2p–Fe 3d transitions.^53^

### 3.8 Optical Property

**Figure 5:**
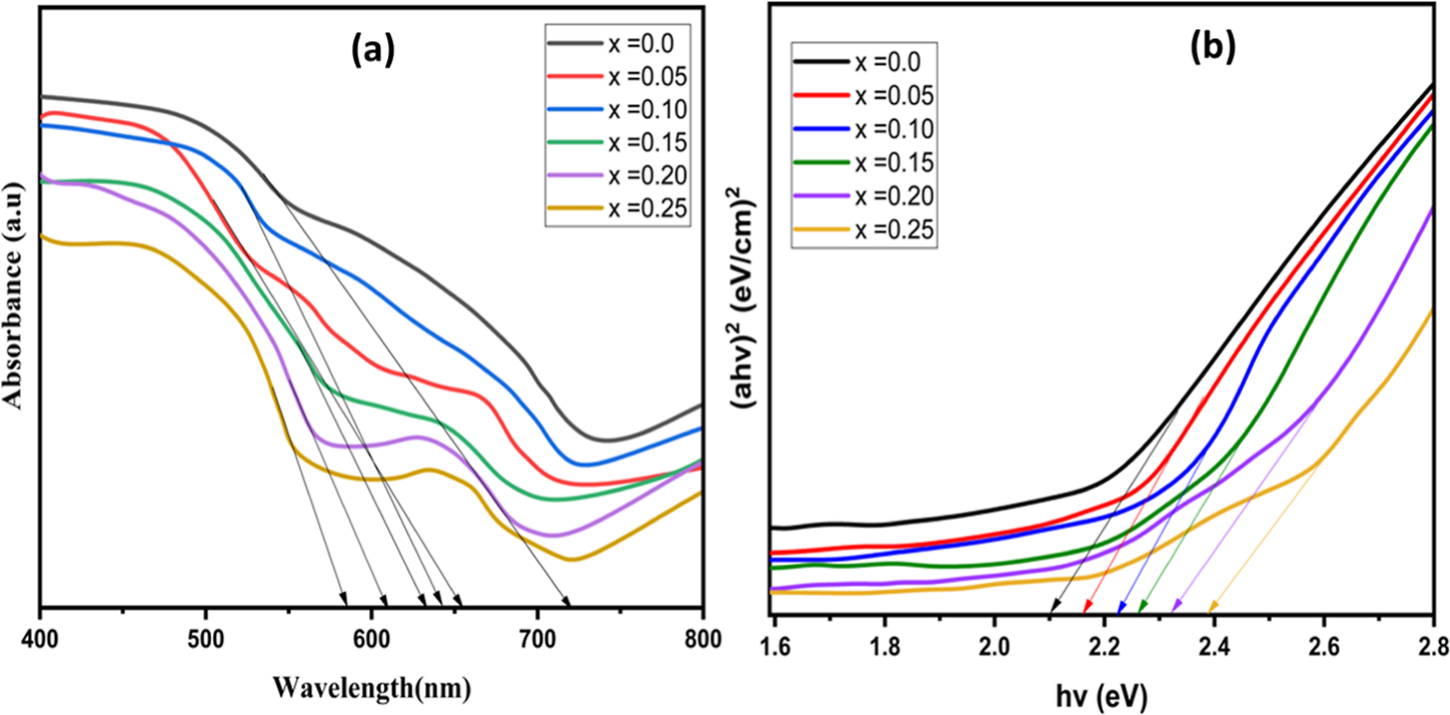
(a) UV spectra and (b) Tauc plots of various compositions of BiGa_*x*_Fe_1–*x*_O_3_.

Using UV–visible spectroscopy, the band gaps of BFO and BiGa_*x*_Fe_1–*x*_O_3_ samples are investigated. The findings for samples of BFO and BiGa_*x*_Fe_1–*x*_O_3_ are depicted in Figure 5. The absorption edges of substituted materials can be seen to be shifted to the left when compared to those of undoped BFO. Tauc plot examination of the spectroscopic data confirms that these changes indicate band gap broadening in the doped compounds, which occurs at lower energy wavelengths of light. Tauc plots are constructed using the UV–visible data (eq 13).
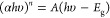
13

The band gap was appraised using α (absorption coefficient), λ (wavelength), *h* (Planck’s constant), *C*′ (proportionality constant), υ (frequency of light), and *E*_g_ (band gap energy). To determine the band gap, we plot (αhυ)^2^ against hυ and use eq 12 to extrapolate the linear portion of the graph until it intersects the *x*-axis.^8^ The band gap values for BFO and BiGa_*x*_Fe_1–*x*_O_3_ are shown in Figure 5, along with the dimensions of the crystallites to observe the influence of the crystal size on band gaps.

The BiGa_*x*_Fe_1–*x*_O_3_ sample has a larger band gap than BFO due to alterations in electronic band structure, particularly in the Fe 3d state. The fact that the ligand field transition wavelength increased by increasing Ga content indicates an increase in the energy required to split the crystal field.^53^ Furthermore, it is noted that in the BiGa_*x*_Fe_1–*x*_O_3_ system, the Ga/Fe ratio is not the only factor that governs the electrical, optical, and magnetic properties. Other factors such as symmetry alterations, cation occupancy disorder, and O_2_ vacancies also play a significant role.^53,54^ When oxygen ions hop between Fe^2+^ and Fe^3+^, charge trap levels can be generated in the energy band gap, particularly when the number of oxygen vacancies is high. It was also found that the ratio of Fe^2+^ to Fe^3+^ states in GFO varies with Fe/Ga cation concentration.^53^ Therefore, the rise in band gap with rising Ga content is a result of the existence of a significantly larger number of Fe^2+^ states in Fe-rich samples or the rise in oxygen vacancies with increasing Ga concentration.^55^ Quantum mechanical effects caused by low-dimensional crystallites are responsible for the expansion of band gaps in nanomaterials. At these length scales, overlapped energy levels disperse and become quantized, causing a widening of the band gap in the material. Thus, the relative low band gap energy of BiGa_*x*_Fe_1–*x*_O_3_ facilitates the photocatalytic activity under visible light.

### 3.9 Photocatalytic Potential

**Figure 6:**
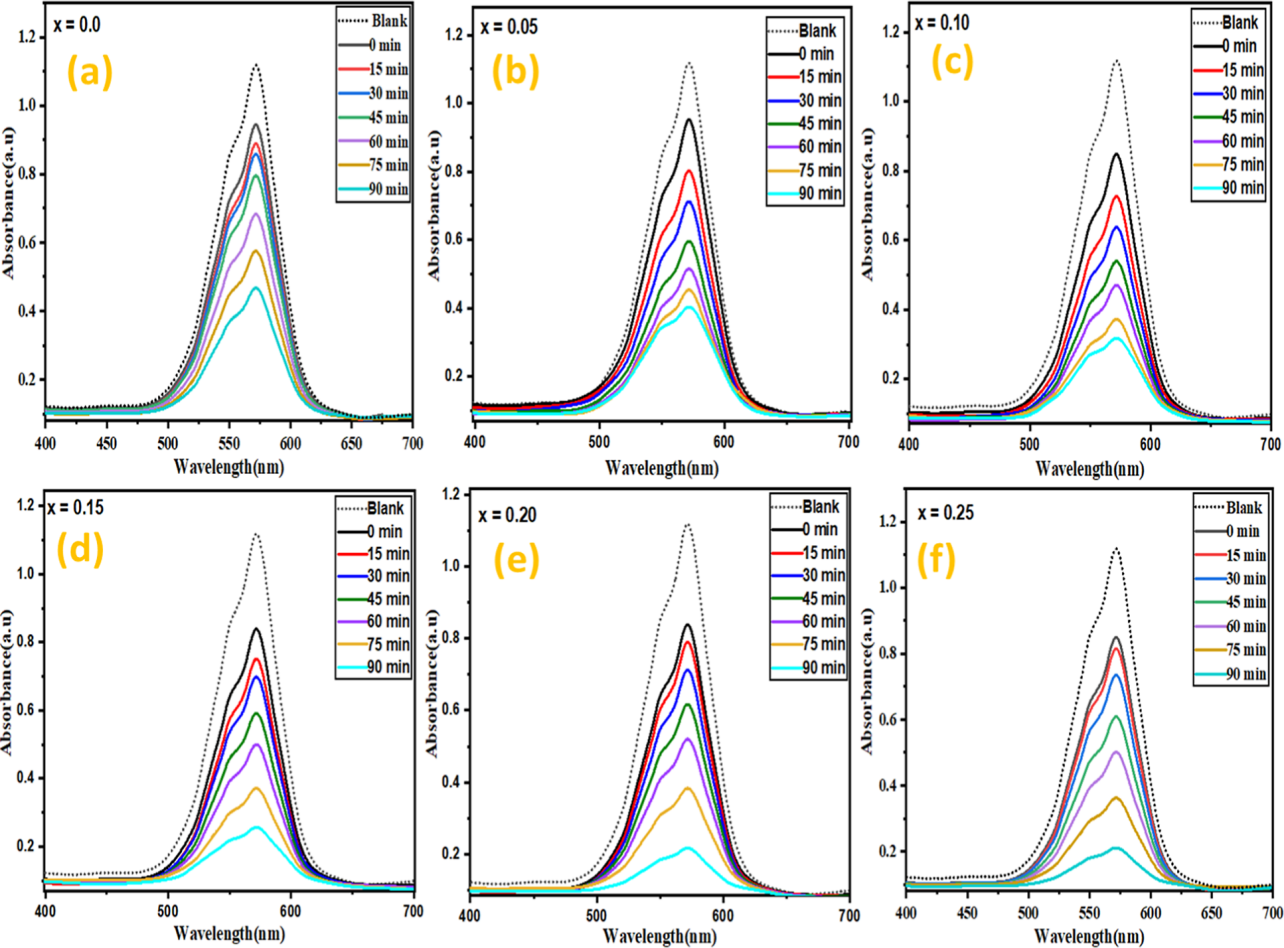
(a–f) UV–vis spectra of CV dye using several concentrations of the dopant content (*x* = 0.0–0.25).

The PCA was estimated and compared by degrading the CV dye under visible irradiation, which is one of the major environmental issues at present.^56–59^Figure 6 exhibits the absorption spectra of dyes in the presence of BiFeO_3_ and BiGa_*x*_Fe_1–*x*_O_3_. The blank absorption of the pure CV dye solution was also recorded to determine the starting concentration of the CV dye. The proportion of the adsorbed CV dye was also evaluated by adsorption/desorption equilibria by measuring the absorbance spectrum (0-time) after agitating the sample solution (CV dye + photocatalyst) in the dark for 1 h. The pristine BFO photocatalyst adsorbs 15% CV dye during the dark process, whereas the BiGa_*x*_Fe_1–*x*_O_3_ photocatalyst adsorbs 25% CV dye. The enhanced adsorption capability of BiGa_*x*_Fe_1–*x*_O_3_ compared to pristine BFO can be attributed to its porous structure and increased surface area, which provides extra CV dye adsorption sites.^60^ The whole process of degradation was carried out for a time period of 90 min for each dye which includes time intervals of 15 min to obtain a sample for absorption study. These absorption spectra validate the high photocatalytic activity of prepared samples as BiGa_*x*_Fe_1–*x*_O_3_ showed a larger intensity drop as compared to BiFeO_3_ for each dye. The outcomes of the photocatalytic study clearly authenticate that the highly doped sample (*x* = 0.25) degraded almost 79% of the dye and the undoped sample depicted only 54% degradation. This upgraded removal efficiency was ascribed to the more number of active sites for maximum adsorption, and tuned band gap may be attributed to this high photocatalytic activity.^61,62^ In comparison to various related photocatalysts, BiGa_*x*_Fe_1–*x*_O_3_ showed promising photocatalytic activity for the degradation of dye (Table 4).

**Table 4:** Comparison of PCAs of Various Catalysts and the Present Study

catalysts	dye	dye removal (%)	reference
BFO/g-C_3_N_4_	methyl orange	75	(63)
Bi_25_FeO_4_0	rhodamine B	30	(64)
Bi_36_Fe_2_O_57_	rhodamine B	9.3	(65)
BiFeO_3_/Citric acid	rhodamine B	72	(66)
BNBFO	methyl blue	75	(67)
BiGa_*x*_Fe_1–*x*_O_3_	CV	79	present work

#### 3.9.1 Plausible Mechanism

**Figure 7:**
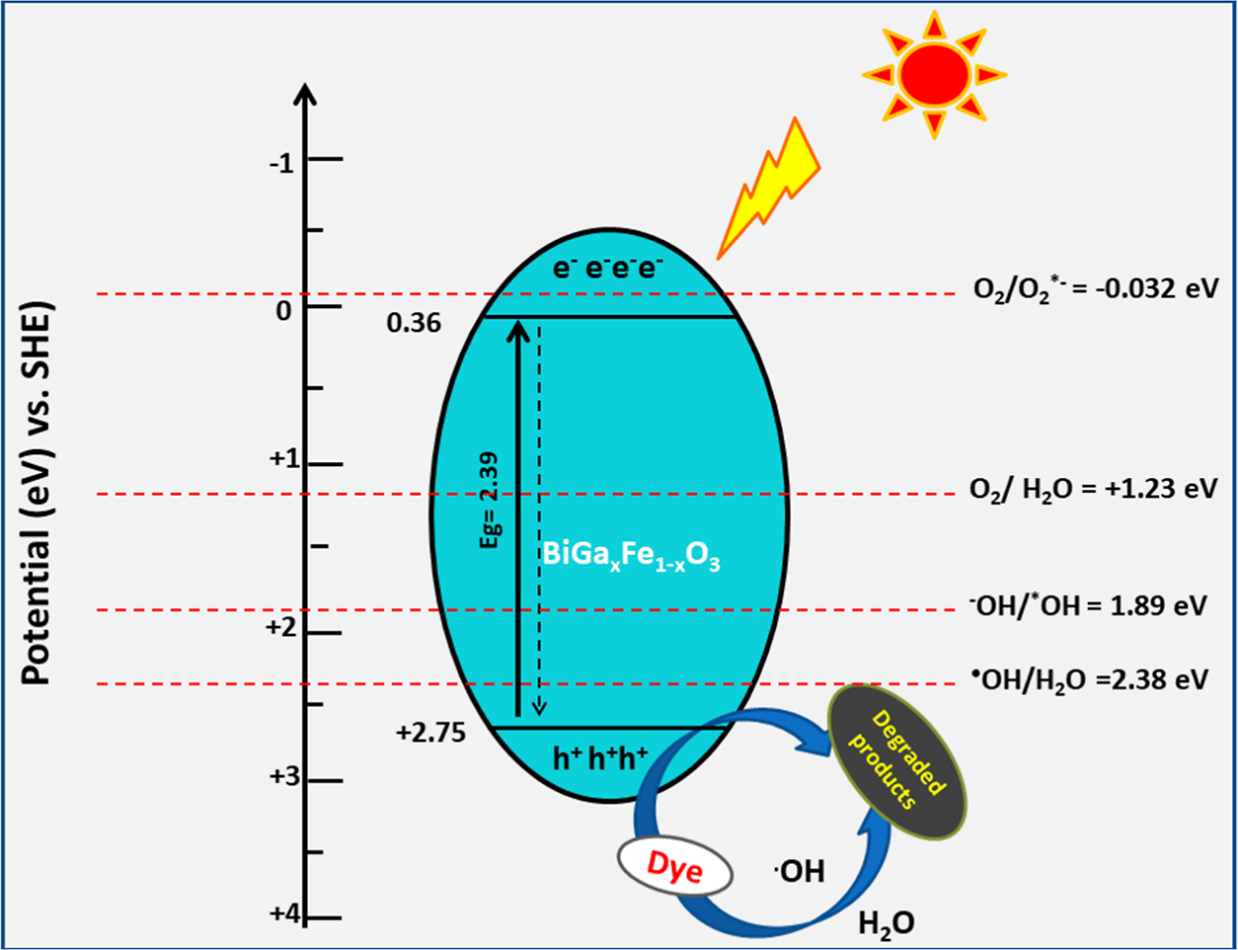
CV dye degradation proposed mechanism using BiGa_*x*_Fe_1–*x*_O_3_ NPs under visible light irradiation.

Figure 7 depicts a potential photocatalytic process for BiGa_*x*_Fe_1–*x*_O_3_ in light of the study mentioned above. Using eqs 14–15, we determine the valence band (VB) and conduction band (CB) potentials at point of zero charge from DFT research.^68^
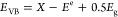
14
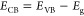
15where *E*_VB_ and *E*_CB_ are respective edge potentials of VB and CB. X represents the geometric average of the electronegativity of atoms that make up the semiconducting material. The hydrogen scale (4.5 eV) value for the free electron energy is represented by *E*^e^, whereas *E*_g_ stands for the energy band gap of the semiconductor. BiFeO_3_ is stimulated by photogenerated electrons (e^–^) and jumps to a high energy state when exposed to light having sufficient energy (>2.5 eV), whereas photogenerated holes (h^+^) stay in low energy state since charge carriers have short life spam in higher energy state; they promptly decay to lower energy and reunite with photogenerated holes, leading to a lower BFO efficacy and a lower number of holes and electrons, as well as influencing the photocatalytic activity.^69^ Electrical interaction among dopants in BFO structure changes the CB edge, increasing the band gap of BFOG from 2.10 to 2.39 eV. This makes it easier to generate excitons at lower energy. As previously stated, replacement causes the creation of shallow ploys in BFOG. Dopants provide shallow traps that collect excited electrons from BFOCE’s VB, leaving h^+^ in VB. h^+^ on VB of BFOG can operate as an oxidant because the VB potential of BFOG is larger than (1.99 eV). In addition, due to the close proximity of the HOMO level of CV and NHE, h + may specifically target CV molecules to cope with the degradation procedure. While photogenic electrons can reduce O_2_ to ^–•^O_2_, the CB potential of BFO and BFOG semiconductors is smaller than the standard oxygen reduction potential (−0.046 eV); therefore, potential cannot be created on CB to reduce O_2_ to ^–•^O_2_. On the other hand, the CB of BFOG lacks adequate negative potential for conversions (−0.046 eV).^70^ On the other hand, holes made in the valence band of BFOG have a adequately positive potential (*E*_VB_ = 2.75 eV) to perform either direct oxidation of dyes or the conversion of H_2_O and –OH to ^•^OH (H_2_O/^–^OH = +1.99 eV vs NHE and –OH/^•^OH = +2.27 eV versus NHE).^71^ Thus, dyes are then broken down by ^•^OH into CO_2_ and water. The classification of BFOG as the photocatalyst with substantial oxidation aptitude is therefore suitable.^72^ The lifespan of photoactive species is increased when dopants are present simultaneously because they effectively stop the e^–^/h^+^ couples from recombining.^73,74^Equations 16–26 provide a summary of the photogenerated species pathway for CV deterioration.^75^ Finding intermediates is difficult because of CV dye’s complex molecular structure. Notwithstanding this constraint, an examination of prior studies permits the formulation of a chemical pathway for the degradation of CV dye, as seen in Figure 7. During the first stage, *N*-demethylation and the chromosphere structure of CV dye are broken down to produce oxidized components. As a result, intermediates undergo hydroxylation and aromatic conjugation is disrupted. These hydroxylated entities then go through an oxidation process to produce quinoid chemicals.^76^ The next step involves the opening of aromatic rings, which results in the formation of carboxylic acids. In the end, additional breakdown processes cause the rings to open, which eventually results in the formation of CO _2_, H _2_ O and inorganic ions.^76^
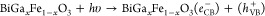
16
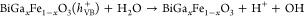
17
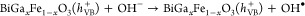
18
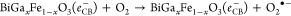
19
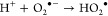
20
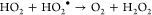
21
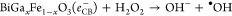
22
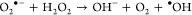
23
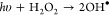
24
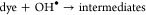
25
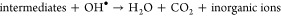
26

#### 3.9.2 Kinetic Study

Kinetic study gives information about the degradation efficiency (*A*_*t*_/*A*_0_) of photocatalysts with respect to time (min). The rate constant (*k*) was appraised by employing eqs 27 and 28.
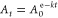
27
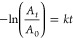
28

In the above equations, *A*_*t*_ is the absorption at time interval, *A*_0_ is the absorption at start, *k* is the rate constant for the reaction, and *t* is the exposure time of sunlight. The plot of *A*_*t*_/*A*_0_ and −ln *A*_*t*_/*A*_0_ versus time for each of the dye has been shown in Figure S8. The photocatalytic reactions mainly obey pseudo-first-order kinetics, as revealed by linear fitting of the graph. The rate constants of BiGa_*x*_Fe_1–*x*_O_3_ (*x*, *y* = 0.0, 0.05, 0.10, 0.15, 0.20, and 0.25) were 0.008, 0.009, 0.01, 0.011, 0.013, and 0.014 (min^–1^), correspondingly. It was observed that BiGa_*x*_Fe_1–*x*_O_3_ (*x*, *y* = 0.25) degraded CV more efficiently than other photocatalysts. The elimination of dye is 54% by BFO (7.48% adsorption +46.52% degradation) and 79.26% by BFOG 5 (6.23% adsorption +73.03% degradation). The efficiency was compared with reported similar studies, and it was perceived that BiGa_*x*_Fe_1–*x*_O_3_ degraded crystal violet more proficiently than other photocatalysts. The rate constant values for each dye immensely prove that the doped sample exhibit higher photocatalytic activity.

### 3.10 Scavenging Studies

The significant purpose of scavenging analysis was to ascertain the most effective agent responsible for the removal of CV dye. Numerous scavengers were consumed in a PCA to scrutinize the function of OH^•^, h^+^, and e^–^. “2-propanol”, “EDTA”, and “AgNO_3_” were used as the trapping agents for ^•^OH, electrons, and holes, respectively. Figure S9 illustrates the kinetic graphs for the CV dye photocatalytic degradation in the presence of scavengers. Scavenging experiments depicted that 2-propanol, silver nitrate, and ethylene-diamine-tetra-acetic acid (EDTA) predominantly reduced the degradation rate, concluding that the primary photoactive species involved in CV dye photodegradation are OH^•^, h^+^, and e^–^. However, the most significant agent for the degradation and the main active species were found to be OH^•^, while the electrons played a negligible role. The active species participating in the photodegradation process were recorded in the following order, electrons < holes < hydroxyl radicals.

### 3.11 Recycling and Stability Studies

For practical applications of the photocatalyst, its photostability is a paramount factor. In order to determine the photostability of BiGa_*x*_Fe_1–*x*_O_3_, reusability experiment was performed regarding degradation of different dyes under visible irradiation, and findings of these experiments are depicted in Figure S10. The photocatalyst was recycled 14 times, and after each cycle, the catalyst was recovered and subjected to the next cycle. The recovered photocatalyst was then made ready for the next cycle by drying it at 90 °C for a sufficient time. BiGa_*x*_Fe_1–*x*_O_3_ exhibited a CV degradation efficiency of 79–63.1% from first to 14th runs in 180 min. The results after 10 cycles of reusability exhibit significant changes in degradation efficiency of BiGa_*x*_Fe_1–*x*_O_3_ for CV dye after multiple cycles. This decline may be attributed to the leaching of the photocatalyst, aggregation of particles, and unavailable surface area.

### 3.12 Effect of pH

The significant degradation efficacy of BiGa_*x*_Fe_1–*x*_O_3_ was confirmed by the rapid removal of CV from the aqueous medium. The pH of the solution is a significant parameter in dye degradation because it can affect the surface charge of catalysts, the degradation tendency of the photocatalyst, due to its effect on functional groups, degree of ionization, and structure of dye molecules.^77^ The CV dye degradation behavior of BiGa_*x*_Fe_1–*x*_O_3_ was further investigated using an experimental setup with pH values ranging from 4 to 10 (Figures S11 and S12). The solution pH was controlled by using 0.1 M HCl and NaOH solutions at room temperature. The effect of pH on the degradation rate of CV by BiGa_*x*_Fe_1–*x*_O_3_ was studied for this purpose. The complete degradation experiment was conducted at pH ∼ 7, and at this pH, BiGa_*x*_Fe_1–*x*_O_3_ exhibited the 79.26% degradation rate for CV. When pH was increased from 4 to 10, the degradation rate of CV increased from 79.26 to 83.3% (pH 10). The degradation efficiency of the cationic dye CV is observed to be decreased in acidic conditions, with a significant drop in degradation behavior. With increasing pH, the surface of the catalyst becomes negatively charged by adsorbed hydroxyl ions; thus, the presence of large quantities of adsorbed OH^–^ ions on the surface of the catalyst promotes the formation of OH^•^ radicals, and thus, the rate of photocatalytic degradation increases.^78^

### 3.13 Antibacterial Activity

**Figure 8:**
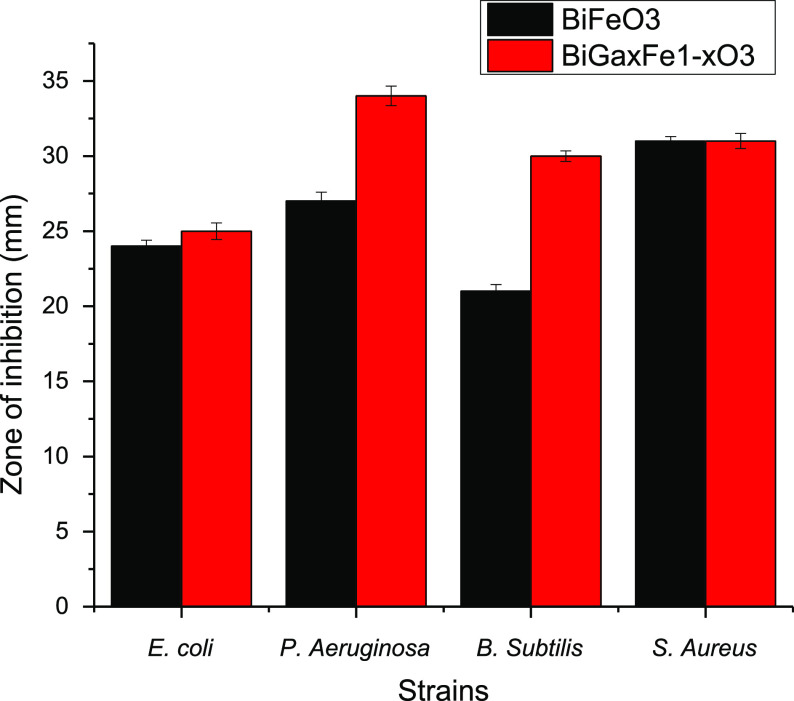
Antibacterial activity for pure and doped bismuth ferrite against a panel of selected microbes.

Both undoped BiFeO_3_ and doped BiGa_*x*_Fe_1–*x*_O_3_ NPs have been tested for their antibacterial efficacy against *Pseudomonas aeruginosa*, *Escherichia coli*, *Staphylococcus aureus*, and *Bacillus subtilis* bacterial strains. The zones of inhibition are depicted in Figure 8 for pure and Ga-doped bismuth ferrite values against various bacterial strains. The doped BiGa_*x*_Fe_1–*x*_O_3_ NPs showed significant antibacterial action against *P. aeruginosa*, with a ZI value of 34 mm, respectively. Meanwhile, in the case of Gram-positive bacteria, doped materials demonstrated less antibacterial activity values versus Gram-negative bacteria.

The fact that both Gram-negative and Gram-positive bacteria are negatively charged cells that support electrostatic contact with NPs or liberate ions from them further suggests that fabricated NPs possess the ability to kill both types of bacteria. Despite the fact that Gram-negative bacteria are more susceptible to BiGa_*x*_Fe_1–*x*_O_3_ NPs, this is due to chemical and structural differences between Gram-negative and Gram-positive bacteria, which are distinguished by the latter’s outer coat of lipopolysaccharides rather than peptidoglycan. Lipopolysaccharide is less stiff than peptidoglycan that can be readily broken.^79,80^ Although the precise mechanism behind the antibacterial action of BiGa_*x*_Fe_1–*x*_O_3_ NPs is unknown. The current study’s experimental results confirm that BiGa_*x*_Fe_1–*x*_O_3_ NPs may trigger intracellular leakage of materials in all examined bacteria. Consequently, we hypothesize that ferrites’ antimicrobial mechanism involves the generation of free radicals, particularly reactive oxygen species, via the Fenton reaction,^81–83^ and creates oxidative stress on bacteria. This causes bacterial cell membrane rupture and the leaking of cytoplasmic materials, which results in a loss of metabolism and cell death. This research is the first of its type to look at membrane leakage caused by BiGa_*x*_Fe_1–*x*_O_3_ NPs.

Bismuth ferrites with Ga doping have demonstrated encouraging activity for *P. aeruginosa*. Clinical *P. aeruginosa* strains have been successfully stopped from growing when treated with BiGa_*x*_Fe_1–*x*_O_3_. Gallium compounds’ capacity to mimic iron is correlated with their antibacterial effects. Due to their similarity to Fe(III) ions, Ga(III) ions can interfere with bacterial iron metabolism by substituting for Fe(III). Usually, the bacteria’s ferric reductases convert Fe(III) to more soluble ferrous ion Fe(II) for inclusion in iron-dependent enzymes. Iron-dependent enzymes cannot convert Ga(III) incorporated into them to Ga(II), which prevents the enzyme from working. Because iron is a crucial micronutrient for the development, survival, and virulence of many bacteria but is difficult to acquire in a mammalian host, interference with iron metabolism in bacteria has been shown to be successful at reducing infection rates. So, using substances based on gallium that prevent bacteria from using iron may have positive antibacterial effect.^84^

## 4 Conclusions

Ga-doped BiFeO_3_ NPs with varied compositions were prepared by a microemulsion route, and the effect of doping was appraised based on structural, dielectric, ferroelectric, and photocatalytic properties. XRD, scanning electron microscopy, Raman, and UV data revealed nanomaterials with a distorted rhombohedral crystalline phase with an average crystallite size in the range of 29–34 nm. By increasing the amount of dopant (Ga), the PL intensity of substituted NMs was shown to increase. The polarization of ferroelectric loops with substituted compositions decreased when the doping concentration was increased. An increase in the frequency of the applied voltage resulted in a decrease in the dielectric constant. Under visible irradiation, the BiGa_*x*_Fe_1–*x*_O_3_ photocatalyst degraded CV dye significantly more effectively than BiFeO_3_. The OH^•^ radical was observed as the most active species in the photodegradation process of CV dye. Moreover, the fabricated BiGa_*x*_Fe_1–*x*_O_3_ material showed excellent antibacterial activity against *S. aureus* bacterial strains in comparison to pristine BFO. Due to the promising photocatalytic and antimicrobial activities, BiGa_*x*_Fe_1–*x*_O_3_ could be utilized in photovoltaic devices and photocatalysts for different applications. The photocatalytic activity under solar light makes this material viable for the treatment of wastewater economically on an industrial scale.

## Supporting Information Available

Electrical, ferroelectric, dielectric, conductivity, resistivity, and photoluminescence properties of BiGa_*x*_Fe_1–*x*_O_3_; dye degradation kinetics plots, scavenging studies, recyclability, and reusability data of BiGa_*x*_Fe_1–*x*_O_3_; and effect of pH and irradiation time on CV dye degradation (PDF)
