# Supplementary material for: Optical, Dielectric, Magnetic, Photocatalytic, and Antibacterial Properties of Ga-Doped BiGaxFe1–xO3 Synthesized by the Microemulsion Approach
Source: ACS Omega. 2023 Dec 16;9(1):545–58. doi: 10.1021/acsomega.3c06132 (PMC10785324; doi:10.1021/acsomega.3c06132)
Supplement: Supplementary file 1 — ao3c06132_si_001.pdf [file ao3c06132_si_001.pdf]

Optical, dielectric, magnetic, photocatalytic and antibacterial properties of Ga doped $BiGa_xFe_{1-x}O_3$ synthesized by microemulsion approach

Zarish Nazeer[1], Ismat Bibi[1*], Farzana Majid[2], Shagufta Kamal[3], Norah Alwadai[4,*], Muhammad I. Arshad[5], Adnan Ali[5], Shazia Nouren[6], Maryam Al Huwayz[4], Munawar Iqbal[7,*]

[1]Institute of Chemistry, The Islamia University of Bahawalpur, Bahawalpur, Pakistan
[2]Department of Physics, University of the Punjab, Lahore, Pakistan
[3]Department of Biochemistry, Government College University, Faisalabad, Pakistan
[4]Department of Physics, College of Sciences, Princess Nourah bint Abdulrahman University, P.O. Box 84428, Riyadh 11671, Saudi Arabia
[5]Department of Physics, Government College University Faisalabad, Pakistan
[6]Department of Chemistry, Government College Women University Sialkot, Pakistan
[7]Department of Chemistry, Division of Science and Technology, University of Education, Lahore, Pakistan
*Corresponding Authors: munawar.iqbal@ue.edu.pk (MI), drismat@iub.edu.pk (IB), nmalwadai@pnu.edu.sa (NA)

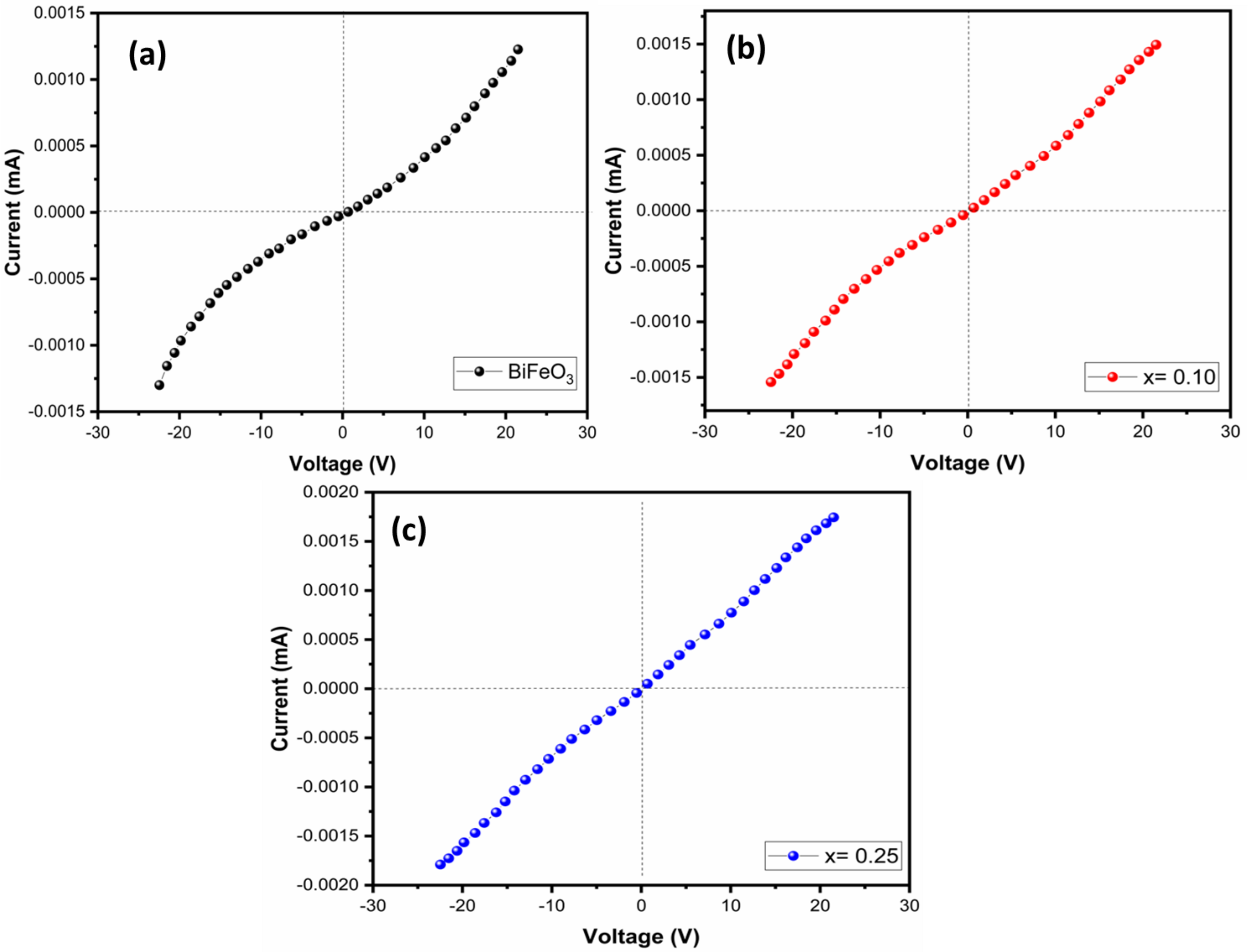

Figure S1: Current voltage (I-V) curves of Ga doped $BiFeO_3$ NPs

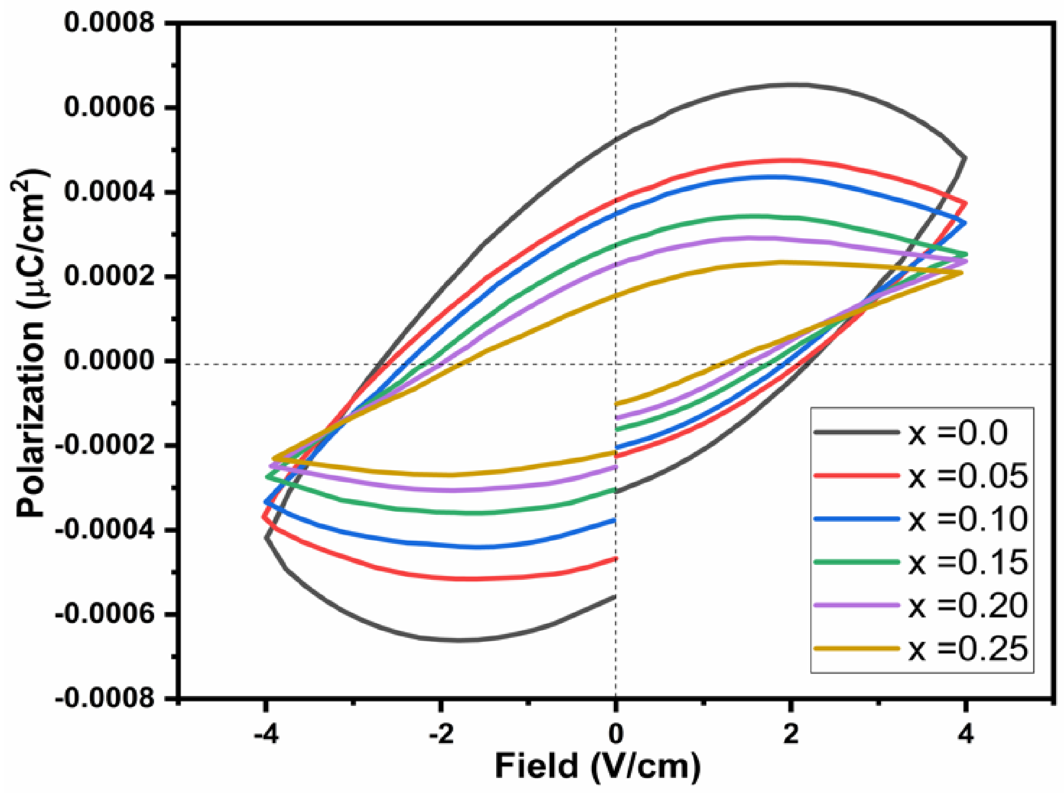

Figure S2: P-E Hysteresis loop of $BiGa_xFe_{1-x}O_3$ measured at 298 K.

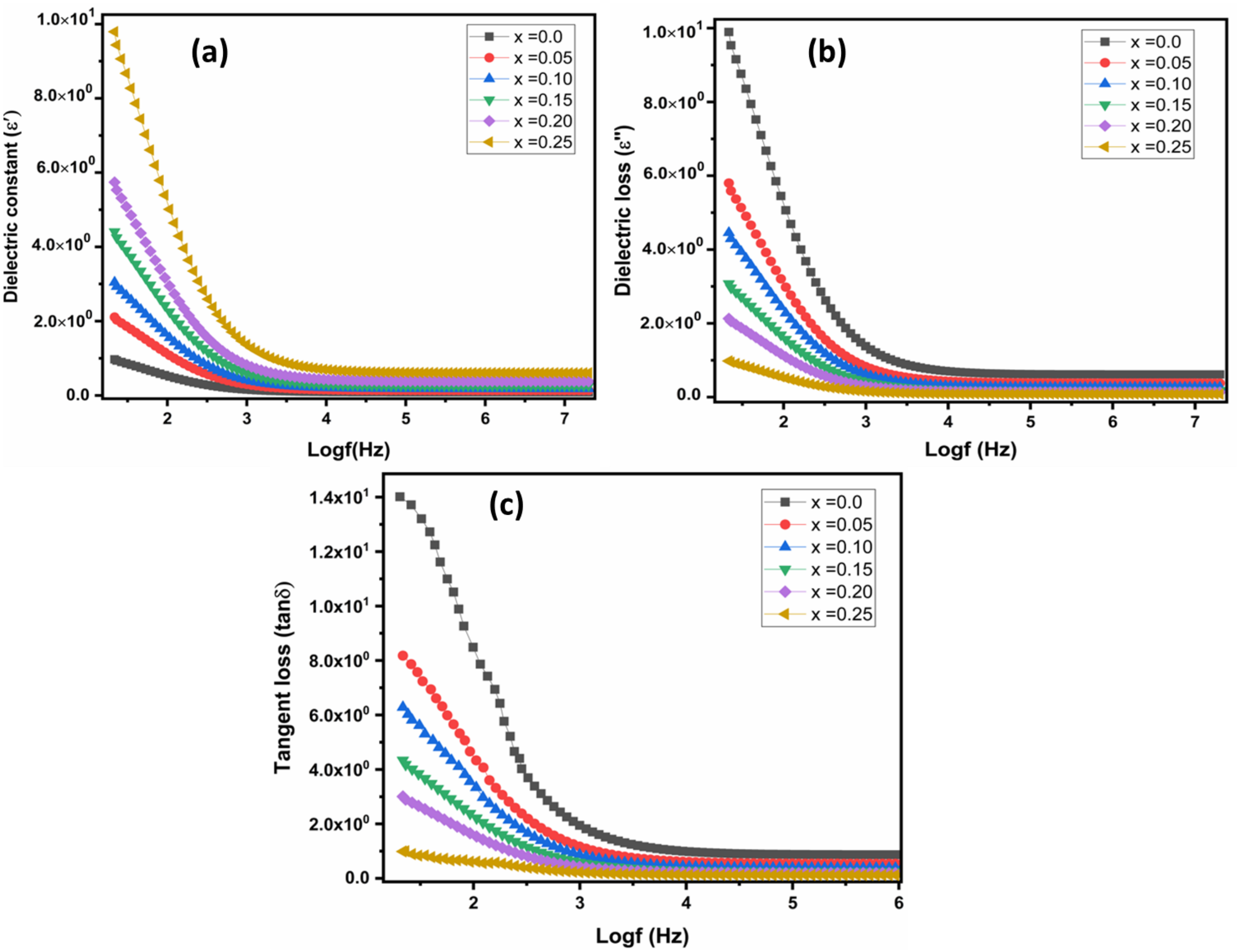

Figure S3: (a) Variation in the dielectric constant of $BiGa_xFe_{1-x}O_3$ as function of frequency (b) Dielectric loss vs. frequency (c) Tangent loss vs. frequency

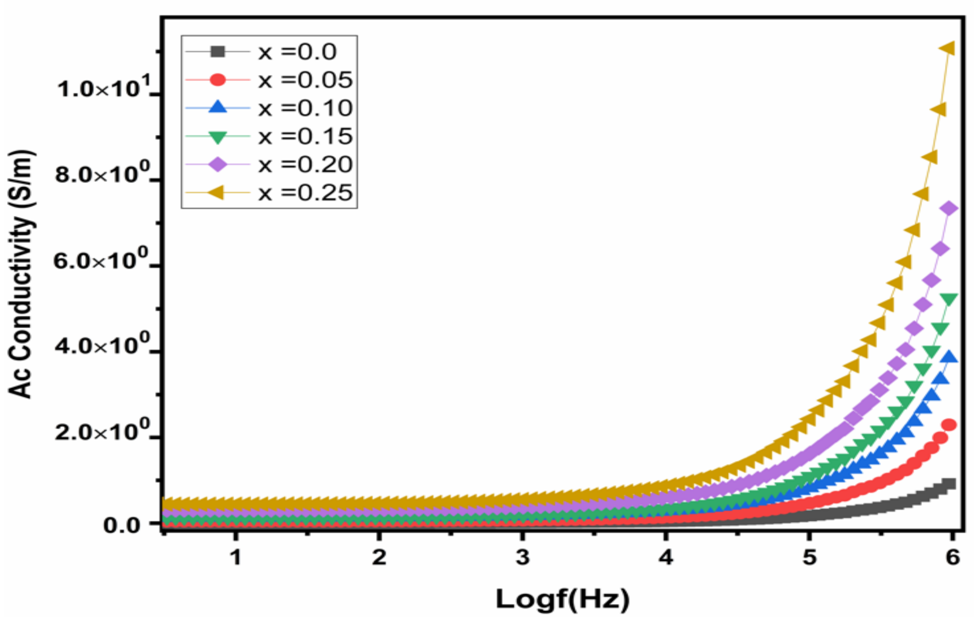

Figure S4: (a) Variation in conductivity of $BiGa_xFe_{1-x}O_3$ as a function of frequency

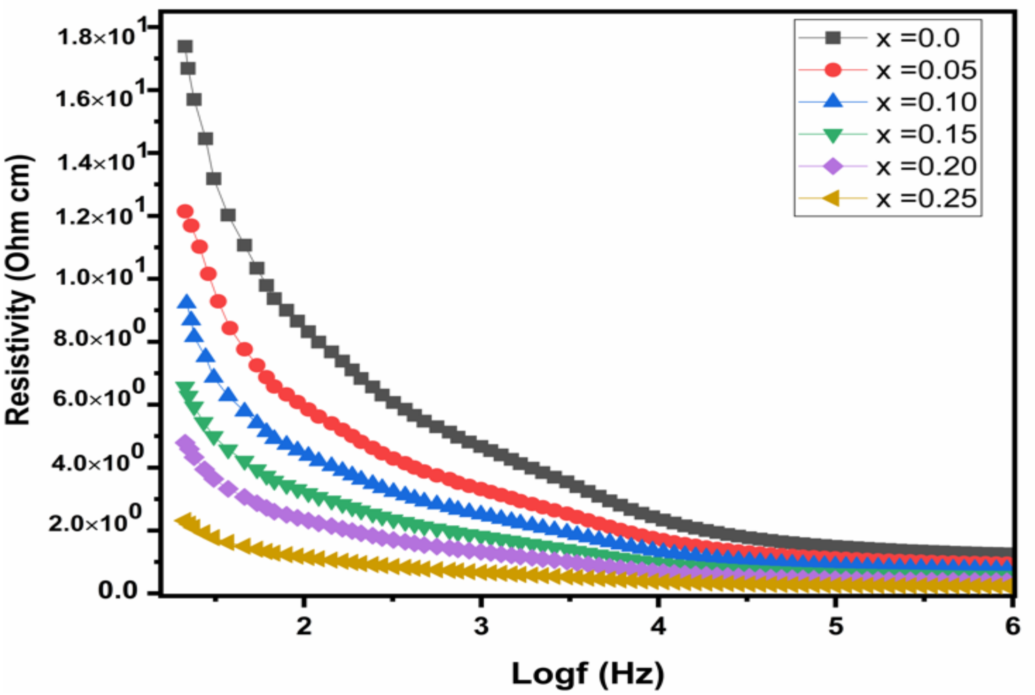

Figure S5: (a) Variation in resistivity of $BiGa_xFe_{1-x}O_3$ as a function of frequency

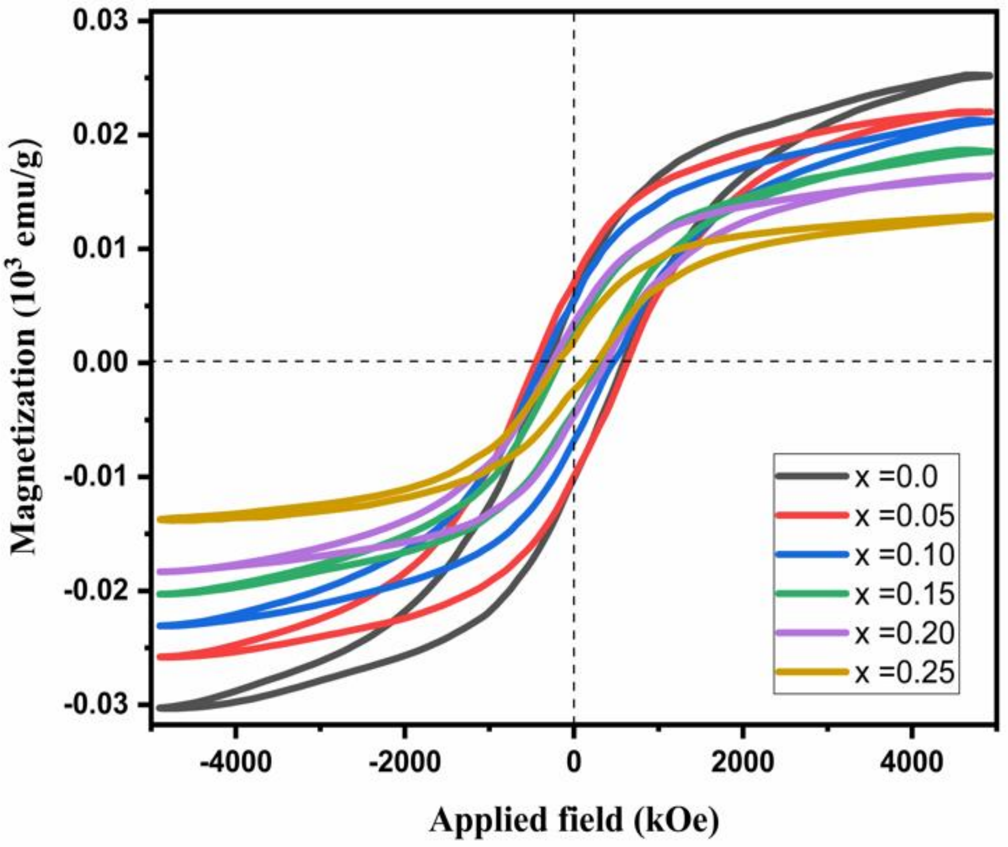

Figure S6: M-H loops of $BiGa_xFe_{1-x}O_3$ NPs

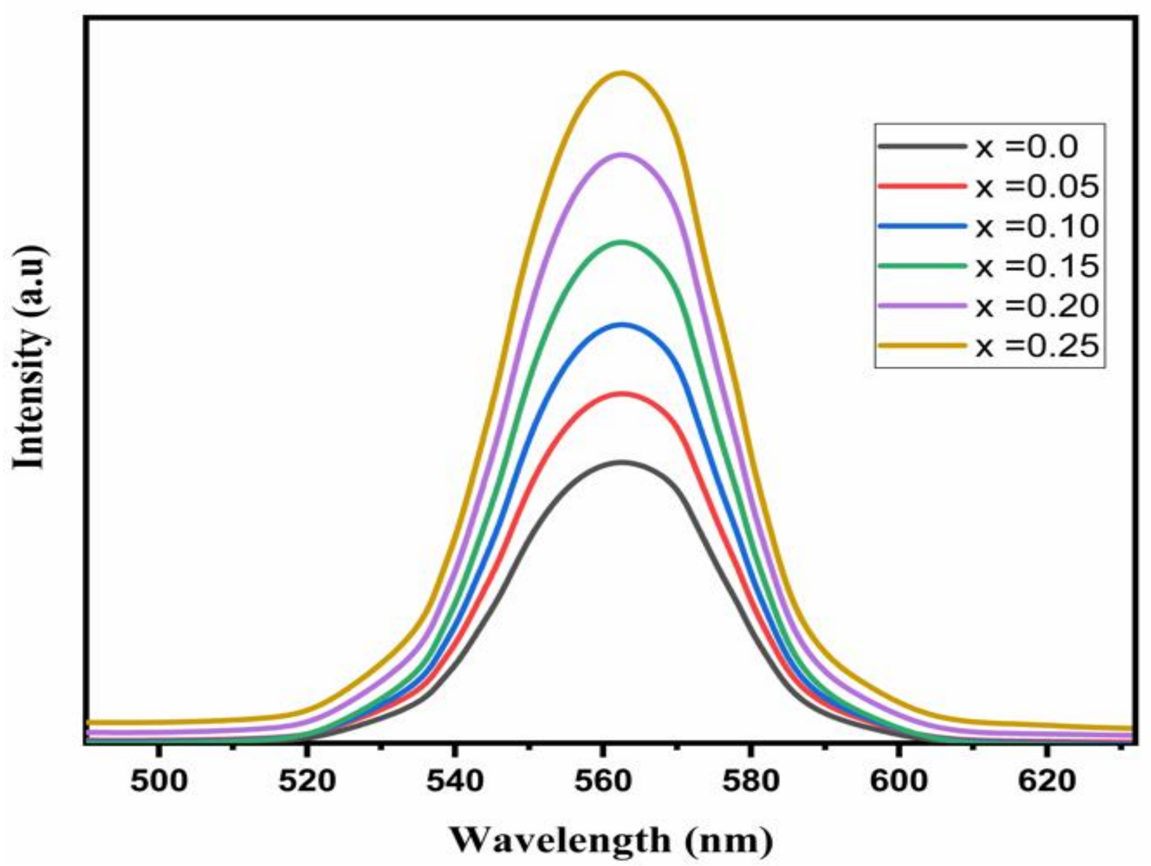

Figure S7: PL spectra of $BiGa_xFe_{1-x}O_3$ NPs.

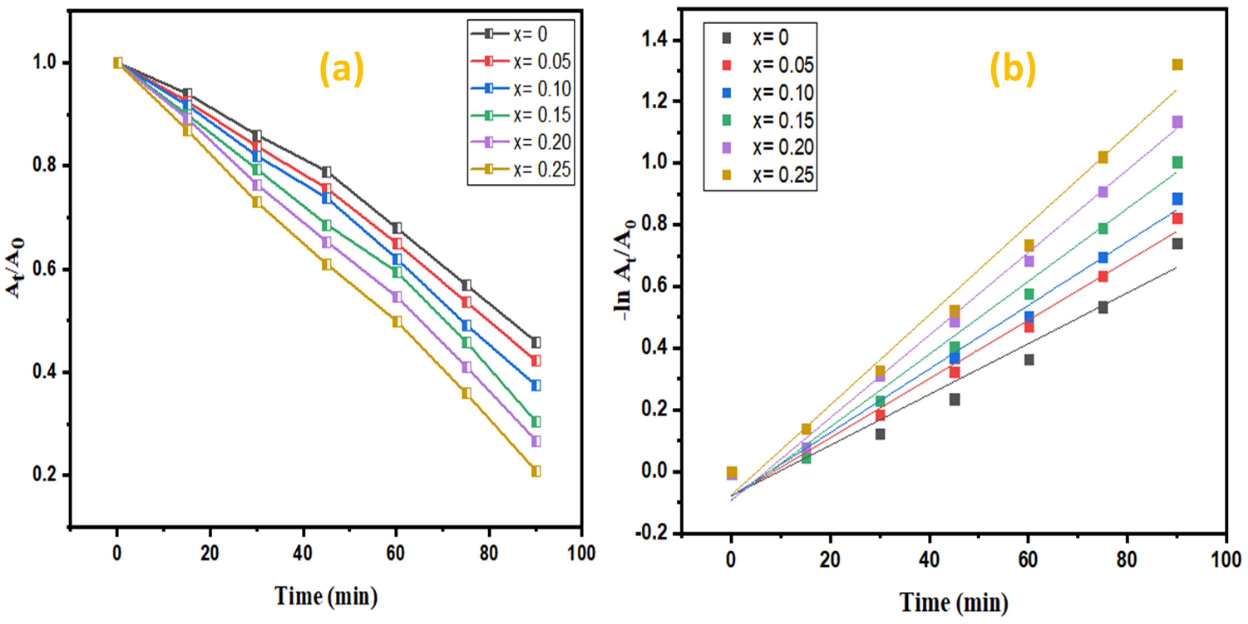

Figure S8: Dye degradation kinetics plots

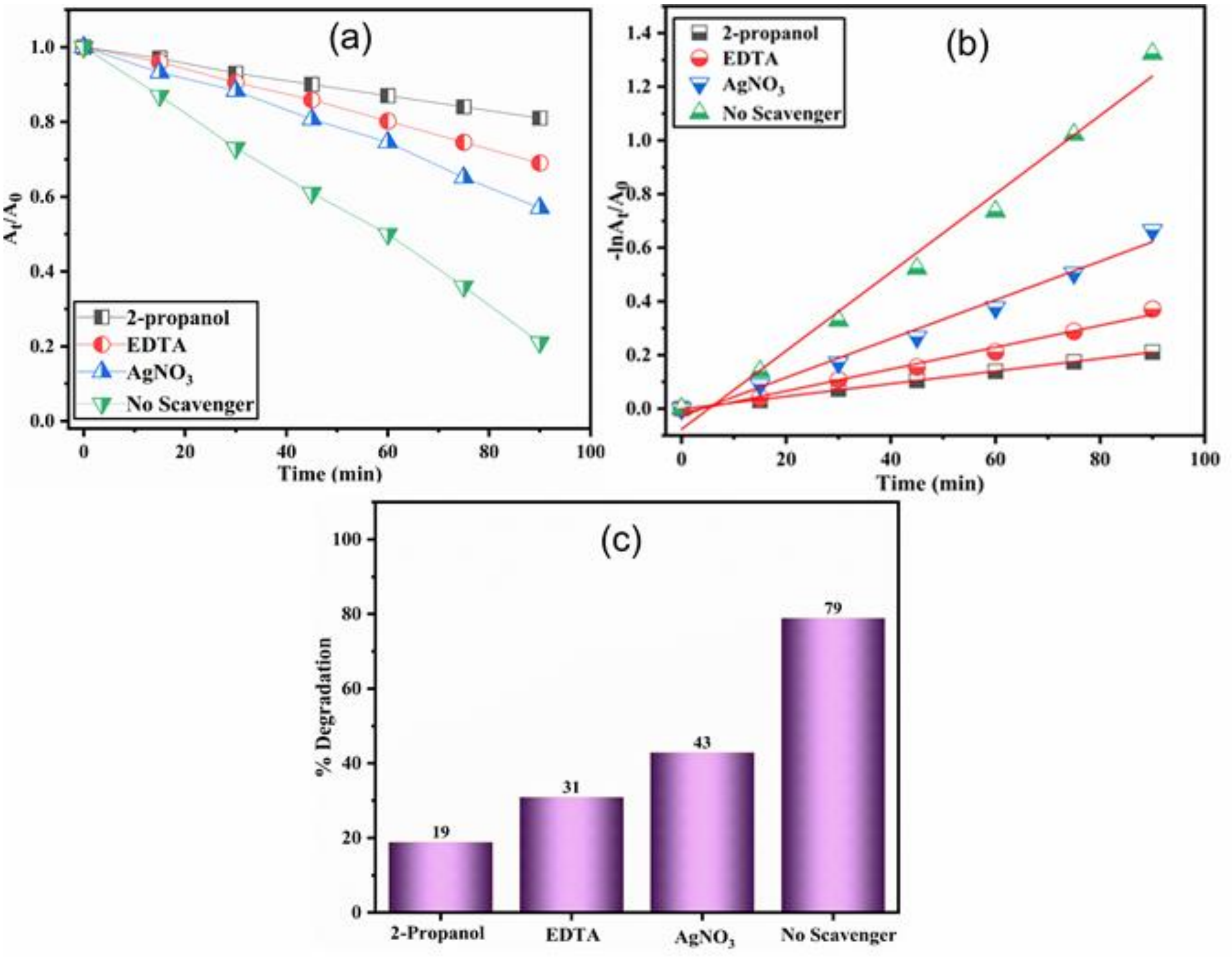

Figure S9. (a-b) kinetics plots in the presence of scavengers and (c) (%) degradation

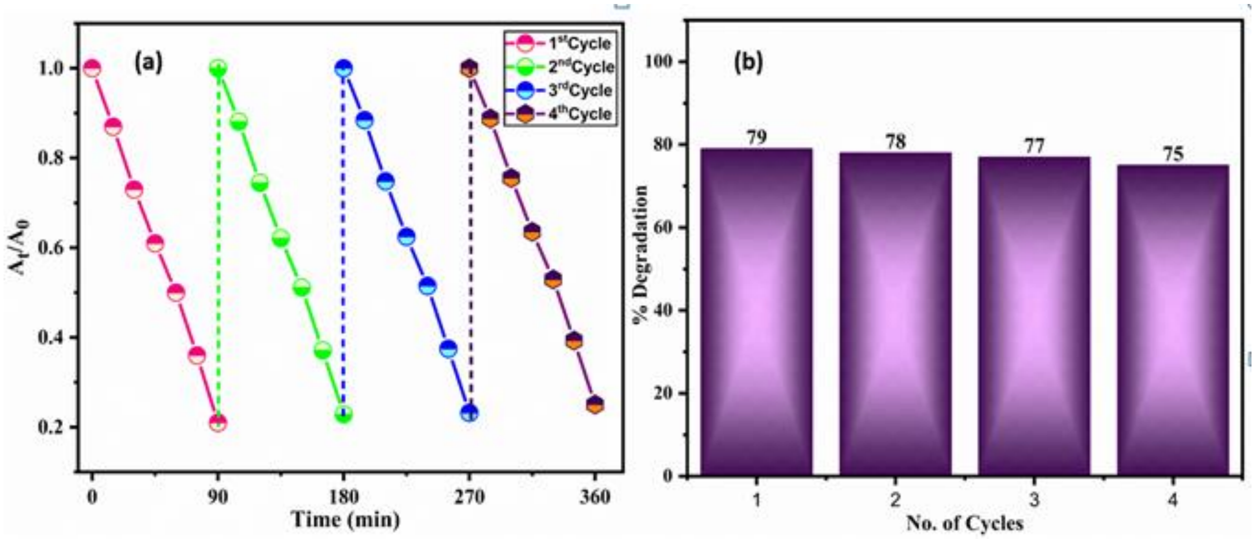

Figure S10. (a) Dye degradation kinetics of four different cycles and (b) Percentage degradation of CV by $BiGa_xFe_{1-x}O_3$ for four different cycles

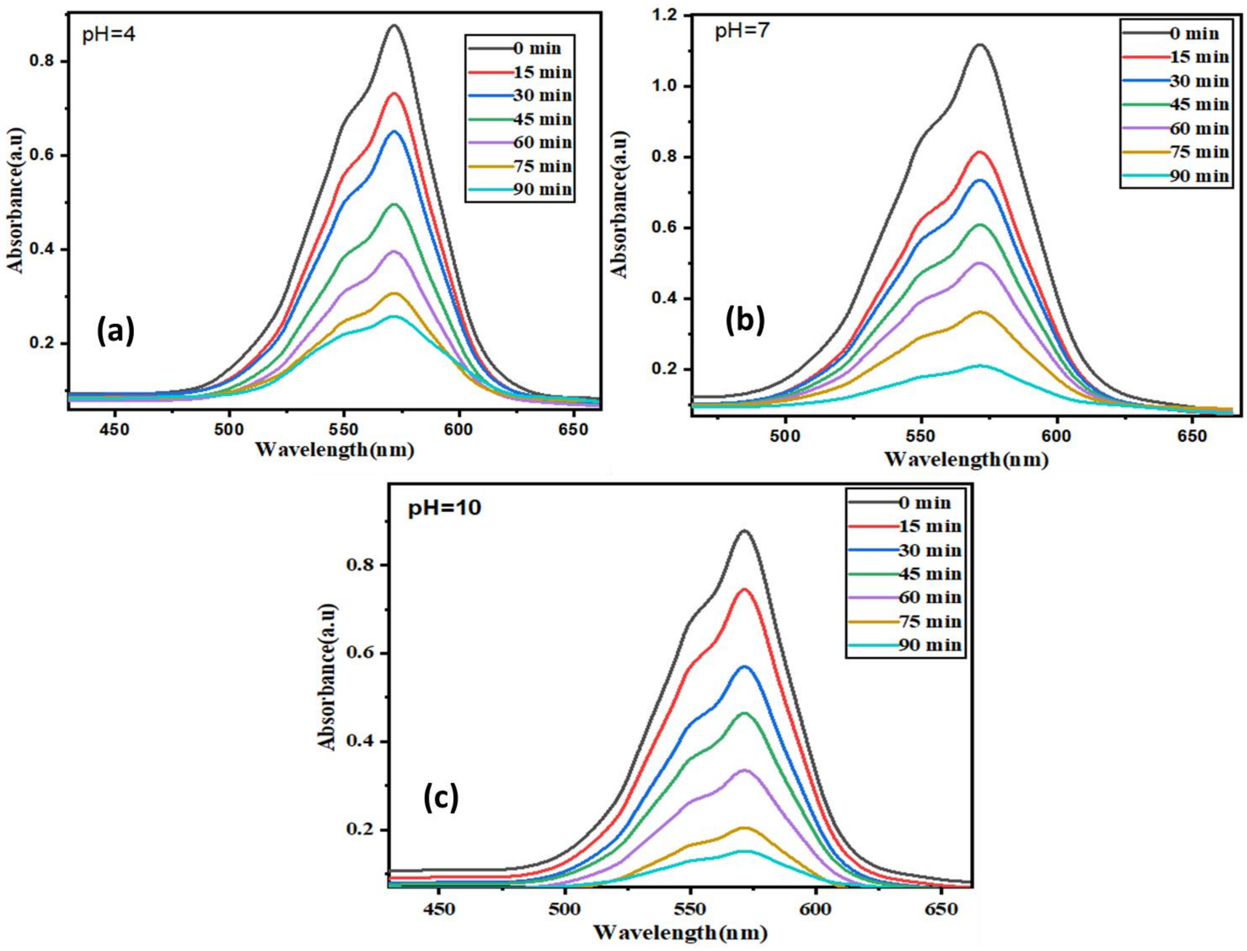

Figure S11: (a-c) UV-vis spectra of CV at different pH using $BiGa_xFe_{1-x}O_3$ photocatalyst

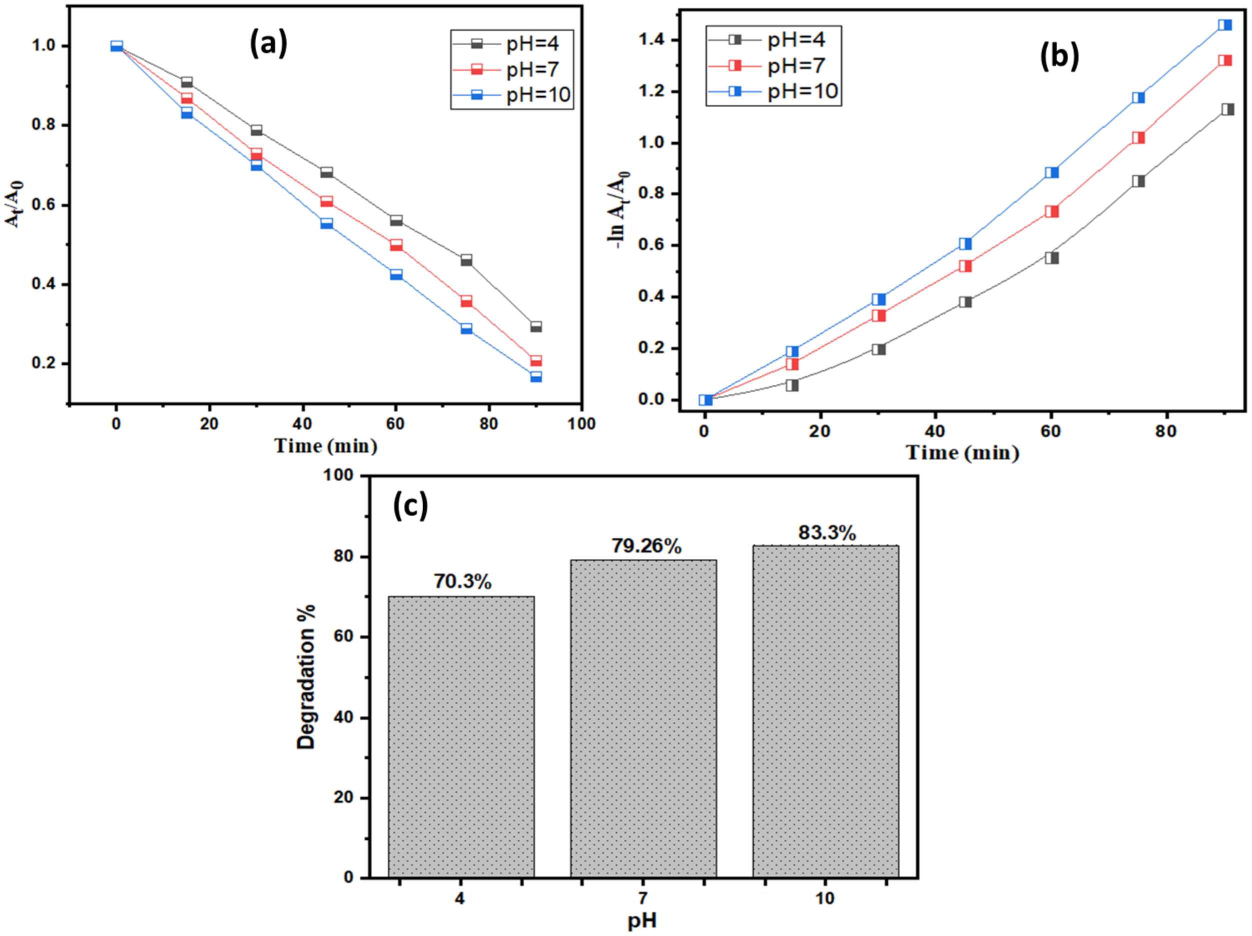

Figure S12: (a, b) A_t/A_o versus time plot of CV dye degradation at different pH and (c) % degradation of CV at different pH values
